# Sex differences in cardiac recovery and ventricular gene expression in a rat model of donation after circulatory death

**DOI:** 10.1186/s13293-026-00915-8

**Published:** 2026-05-23

**Authors:** Anja Helmer, Alexia Clavier, Maria Arnold, Adrian Segiser, Heidi E. L. Lischer, Selianne Graf, Mojgan Masoodi, Georgia Beer, Rahel Ottersberg, Manuel Egle, Matthias Siepe, Sarah Longnus

**Affiliations:** 1 Department of Cardiac Surgery, Inselspital, Bern University Hospital, University of Bern, Bern, Switzerland; 2 Department for BioMedical Research, University of Bern, Bern, Switzerland; 3 Graduate School of Cellular and Biomedical Sciences, University of Bern, Bern, Switzerland; 4 Interfaculty Bioinformatics Unit, University of Bern, Bern, Switzerland; 5 Swiss Institute of Bioinformatics, Lausanne, Switzerland; 6 University Institute of Clinical Chemistry, Inselspital, Bern University Hospital, University of Bern, Bern, Switzerland

**Keywords:** Heart transplantation, Donation after circulatory death (DCD), *Ex vivo/ex-situ* heart perfusion, Cardioprotection, Sex as a biological variable (SABV), Bulk RNA Sequencing (RNA-Seq.), Transcriptomics

## Abstract

**Background:**

Heart donation after circulatory death (DCD) is a promising strategy to increase graft supply. However, in contrast to conventional heart transplantation, in which organs are retrieved from heart-beating donors, DCD hearts are subjected to damaging conditions before and during functional, warm *in-situ* ischemia in the donor, leading to ischemia-reperfusion injury (IRI). Although sex differences have been identified in other contexts of cardiac IRI, such as myocardial infarction, they remain underexplored in DCD. Therefore, we aimed to investigate whether sex differences induce changes in the expression of genes in response to cardiac DCD conditions, including IRI, which may contribute to sexual dimorphism in graft quality.

**Methods:**

102 animals were included in this study. Male, female, and ovariectomized (OVX) Wistar rats underwent simulated DCD with no or 22 min of functional, warm *in-situ* ischemia, followed by oxygenated reperfusion with left-ventricular loading. Functional recovery was assessed and left-ventricular tissue was used for RNA-sequencing.

**Results:**

Recovery of left ventricular function was decreased by functional, warm *in-situ* ischemia, but significantly better in females than in males, with OVX resembling the males. Reperfusion induced inflammatory, stress-response and metabolic-related pathways in all groups. Expression of 110 genes correlated with cardiac recovery, many of which were more abundant in females compared to males, consistent with a role in improved post-ischemic ventricular function. Among these genes, Igfbp3, Fam78b, and Galnt10 were differentially expressed in females compared to males and OVX, suggesting an influence of female sex hormones.

**Conclusions:**

Compared to male hearts, cardiac recovery is significantly higher in female hearts after exposure to DCD conditions and is accompanied by an increased expression of genes related to quality control programs that positively correlate with ventricular function. Significantly higher expression of genes related to energy metabolism, including fatty acid metabolism, and inflammatory pathways was revealed in males compared to females and is associated with decreased recovery. This study suggests potential new therapeutic targets for optimizing cardiac DCD graft quality, and highlights the importance of underlying sex and sex-hormone differences, e.g. in inflammatory pathways and metabolic adaptations, that should be taken into consideration for the implementation of sex-specific precision therapies.

**Supplementary Information:**

The online version contains supplementary material available at 10.1186/s13293-026-00915-8.

## Highlights

Female hearts recover significantly better compared to male hearts after simulated donation after circulatory death ischemia and reperfusion. Ovariectomized (OVX) hearts recover similarly to males, suggesting a cardioprotective role for female sex hormones.

Reperfusion is associated with a sex-independent upregulation of pro-inflammatory, stress response and metabolic pathway gene expression.

Among hearts evaluated after DCD-induced functional, warm *in-situ* ischemia and reperfusion, all genes that correlated positively with cardiac recovery were more highly expressed in females, whereas genes that correlated negatively with cardiac recovery were more highly expressed in males and OVX.

A subset of genes was identified as potential novel cardioprotective targets. These genes both correlate with functional recovery and demonstrate differential expression in females compared to both males and OVX, reflecting the pattern of ventricular recovery.

**Supplementary Information:**

The online version contains supplementary material available at 10.1186/s13293-026-00915-8.

## Abstract (plain language summary)

Heart transplantation is currently the only treatment for patients with advanced heart failure to improve quality of life and survival. Nonetheless, the number of newly listed patients needing a transplant continues to increase and outpaces the supply of suitable donors.

One promising approach to increase donor availability is using hearts from donors after circulatory death (DCD). In conventional heart donation, the donor is declared brain-dead, but machines keep the heart beating and oxygenated until removal. In DCD, the heart stops beating in the donor and it briefly receives less oxygen and nutrients, which can cause donor organ injury. Although outcomes with DCD hearts are excellent and transplant rates increase, further optimization of the protocol could allow more hearts to be used.

In other situations where hearts temporarily lack oxygen, such as myocardial infarction, studies suggest that adult female hearts recover better than male hearts. In the DCD setting, little is known, but it has recently been shown in preclinical studies that female hearts are more tolerant to these conditions.

In this study, we simulated the DCD protocol in a rat model. Afterwards, we identified 673 genes that were differentially expressed between male and female hearts. Many of these genes correlated with cardiac recovery; those more abundant in females were linked to better recovery, whereas those more abundant in males were linked to worse recovery. Taken together, our findings may help to identify treatments for the optimization of cardiac graft quality and improve heart transplantation options for both women and men.

**Supplementary Information:**

The online version contains supplementary material available at 10.1186/s13293-026-00915-8.

## Background

Heart transplantation remains the treatment option of choice to improve quality of life and survival for patients with advanced heart failure [1]. Unfortunately, the demand for cardiac grafts is persistently higher than the current offer [2, 3]. One approach to increase graft availability is the use of hearts from donors after circulatory death (DCD). Indeed, several single-centre studies from the United Kingdom and Australia, as well as a multicentre, randomized clinical trial, report similar one to eight-year survival in transplant recipients receiving grafts following conventional donation after brain death (DBD) or DCD [4–7]. Importantly, the implementation of DCD heart transplantation has substantially increased transplant rates in several European countries, as well as the United States and Australia [8]. 

Despite these excellent results, DCD hearts are subjected to potentially harmful conditions and optimized DCD protocols remain to be developed [9]. In contrast to DBD, DCD hearts are procured only after a period of warm *in-situ* ischemia, which raises concern for cardiac injury [10] and may lead to the higher rates of graft discard in DCD compared to DBD [2]. Thus, clinical protocols that minimize DCD-induced graft injury, for example, by including cardioprotective strategies, could help to ensure optimal graft quality and use.

Although very few studies have investigated sex differences in DCD graft recovery, cardiac tolerance to warm ischemia is recognized as sex specific. Indeed, reproductive-age females are more resistant than males, with much evidence coming from studies of myocardial infarction [11–13]. Given that DCD hearts also undergo a period of warm, *in-situ* ischemia before procurement, such differences might likewise influence the quality of DCD grafts. Indeed, Chen et al. recently reported that female rat hearts exhibit smaller infarct sizes and greater mitochondrial integrity compared with males in a DCD setting [14]. These findings align with our recent study, also in a rat model of DCD, which supports a cardioprotective role for female sex hormones [15]. Mechanisms for the superior recovery of cardiac DCD grafts from females remain to be fully elucidated; however, current evidence from other cardiac pathophysiologic conditions indicates a complex interplay between the sex chromosome complement, affecting genes that modulate cardiometabolic traits and chromatin-modifying enzymes, and sex hormones, such as estrogen and progesterone, which help preserve mitochondrial integrity and reduce potentially harmful reactive oxygen species (ROS) generation [16]. Sexual dimorphism extends to metabolic adaptations, with evidence of improved flexibility response to altered substrate availability in females [17, 18]. Given that warm ischemia and reperfusion profoundly alter cardiac energy metabolism, differences in metabolic flexibility may influence DCD graft quality in a sex-specific manner. In addition, female sex hormones have been implicated in exerting anti-inflammatory effects [19, 20]. 

The clinical impact of donor sex differences in DCD heart transplantation outcomes remains to be fully characterized. Limited data availability may result from the relatively low proportion of female DCD donors, which account for only approximately 18–20% of all DCD heart transplants [21]. However, there is evidence that acute rejection is not associated with donor sex in DCD heart transplantation (approximately 13% for recipients of male donors and 16% for recipients of female donors), and that both one- and two- year survival rates are similar for male and female donors, ranging between approximately 92% and 83%, respectively [22]. 

As the feasibility and benefits of DCD heart transplantation have now been demonstrated, we can turn our attention to the optimization of DCD protocols in order to realize its full potential. Given that sex differences in tolerance to DCD conditions have been reported but remain largely unexplored, we investigated differences in gene expression in response to cardiac DCD conditions among males, females and ovariectomized rats in order to identify both sex chromosome and female sex hormone effects. By integrating functional recovery under physiologically relevant loading conditions with transcriptomic changes in the left ventricle at multiple timepoints the DCD protocol, we aim to reveal sex- and sex-hormone- specific differences in the expression of key genes that may serve as novel targets for the application of sex-specific cardioprotective strategies upon graft procurement and during *ex-situ* heart perfusion, thereby contributing to the optimization of clinical DCD protocols.

## Methods

### Animal care

Female, male and ovariectomized female (OVX) Wistar rats were purchased from Janvier Labs (Le Genest-Saint-Isle, France). Ovariectomy was performed at 6 weeks of age. Rats were housed in pairs of two animals per cage under welfare-compliant conditions with a 12-hour light-dark cycle and unlimited access to food and water. Male bedding was added to female cages to stimulate estrous cycling and to OVX cages as a control [23]. Experiments were conducted at 10–12 weeks of age to represent young adult DCD donors.

### Experimental design

**Fig. 1:**
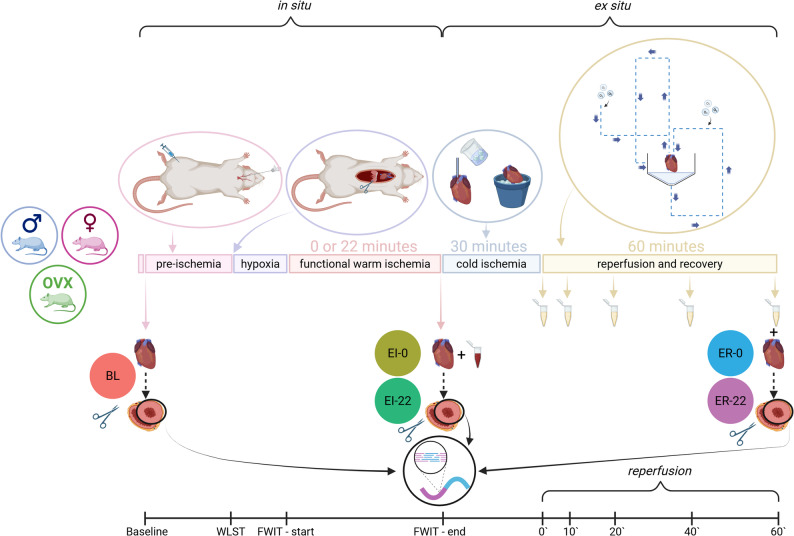
Experimental design: Female, male and OVX rats were randomly assigned to one of five experimental timepoints based on collection and ischemic time: BL (baseline, control timepoint), EI-0 (end-ischemia 0 min timepoint), EI-22 (end-ischemia 22 min timepoint), ER-0 (end-reperfusion 0 min timepoint) or ER-22 (end reperfusion 22 min timepoint). Blood samples were collected at the end of functional, warm *in-situ* ischemia. Perfusate samples were collected at multiple time points during reperfusion. Cardiac recovery was measured during reperfusion. Left ventricular tissue was collected and used for bulk RNA sequencing analysis; WLST, withdrawal of life-sustaining therapy; FWIT, functional warm ischemic time; OVX, ovariectomized; BL, baseline timepoint; EI, end ischemia timepoint; ER, end reperfusion timepoint

The experimental protocol included simulated DCD with either no or 22 min of functional, warm, *in-situ* ischemia, followed by explantation of the heart and *ex-situ* cardioplegic flush, cold storage, and normothermic reperfusion with left ventricular loading. A functional, warm *in-situ* ischemic time of 22 min was chosen based on previous studies to provide an intermediate level of recovery, permitting measurable functional differences, and aligning with ischemic durations that are similar to clinical settings [15, 24–26]. Hearts were harvested at five different experimental timepoints as described in (Fig. 1). Animals were randomly assigned to one of five experimental timepoints: (i) BL (baseline: control hearts harvested immediately after anaesthesia),; (ii) EI-0 (end-ischemia 0 min: hearts collected at functional, warm *in-situ* ischemic time start) (iii) EI-22 (end-ischemia 22 min: hearts collected after 22 min of functional, warm *in-situ* ischemia); (iv) ER-0 (end-reperfusion 0 min: hearts collected after explantation at functional, warm *in-situ* ischemic time start, cardioplegia, cold static storage (CSS) and reperfusion); or (v) ER-22 (end reperfusion 22 min: hearts collected after 22 min of functional, warm *in-situ* ischemia, cardioplegia, CSS and reperfusion). A parallel-arm design was used, with block randomization. 102 animals were included in this study. 19 (6 females, 7 males, 6 OVX) were used as control (baseline) hearts. 23 (6 females, 8 males, 9 OVX) were assigned to the EI-0 timepoint. 19 (5 females, 7 males, 7 OVX) rats were included in the EI-22 timepoint. 21 (8 females, 6 males, 7 OVX) were assigned to the ER-0 timepoint. 20 (6 females, 7 males, 7 OVX) were included in the ER-22 timepoint. Left ventricular tissue was snap-frozen and used for bulk RNA sequencing. Additional omics analyses (phosphoproteomics and metabolomics) have been performed on hearts included in this study. We will report the results of these additional analyses in separate manuscripts in order to clearly report all the relevant methods for reproducibility and to enable a full and fair interpretation of the data.

### *In-situ* experimental procedures

*In-situ* experimental procedures followed our previously published protocol, with minor adjustments to incorporate additional experimental time points [15]. In short, anaesthesia comprising ketamine (Narketan 10, Vetoquinol, Bern, Switzerland) (65.0 mg/kg for females and OVX and 87.7 mg/kg for males), xylazine (Xylapan, Vetoquinol, Bern, Switzerland) (6.0 mg/kg for females and OVX and 8.1 mg/kg for males) and acepromazine (Prequillan; Fatro S.p.A., Bologna, Italy) (1.0 mg/kg for females and OVX and 1.3 mg/kg for males) was administered by intraperitoneal injection. Anesthetic doses were adjusted between sexes to ensure comparable depth of anesthesia and physiological stability across groups, as males required higher doses to achieve and maintain adequate anaesthesia depth. After loss of the pedal reflex, hearts were either directly explanted for the BL timepoint or the right carotid artery was cannulated for intravascular access to monitor blood pressure for all other experimental timepoints. Heparin (Liquemin; Drosspharma, Basel, Switzerland) (750U/kg) was administered through the carotid artery catheter. Oxygen saturation was constantly monitored by foot clip and maintained at 96–99% with oxygen delivered using a nose cone. Withdrawal of life-sustaining therapy (WLST) was simulated by transection of the diaphragm. FWIT was defined to start at systolic arterial pressure of 50 mmHg; EI-0 hearts were directly explanted at this time, while EI-22 hearts were only collected after a period of 22 min of functional, warm *in-situ* ischemia. ER-22 and ER-0 hearts were explanted after a functional, warm *in-situ* ischemic period or not, respectively, following subsequent cardioplegia and normothermic *ex-situ* reperfusion. Functional, warm *in-situ* ischemia was performed under conditions with body temperature maintained between 36.7 and 37.0 °C for 22 min, based on previous studies [24, 27]. 

*In-situ* hemodynamic measurements were monitored and recorded via the cannulated carotid artery with the PowerLab data acquisition system (ADInstruments, Spechbach, Germany).

### *Ex-situ* experimental procedures

Following explantation, ER-0 and ER-22 hearts were flushed via the aorta with cold cardioplegic solution at approximately 4 °C (St. Thomas II supplemented with 83 ng/mL erythropoietin (EPREX, Janssen, Beerse, Belgium) and 100 µg/mL glyceryl trinitrate (Nitroglycerin Bioren, Sintetica SA, Couvet, Switzerland)) and stored on ice at approximately 4 °C in St. Thomas II solution for 30 min (CSS) to simulate clinical workflows, cardioplegic solution contents, including heart explantation, back-table preparation, and priming of the *ex-situ* perfusion system [28–30]. Afterwards, hearts were mounted on an *ex-situ* perfusion system and reperfused for 1 min in Langendorff mode with oxygenated modified Krebs-Henseleit buffer followed by 9 minutes with the addition of 3% bovine serum albumin, 0.5 mM lactate and 0.4 mM palmitate, to simulate physiological substrate availability. Subsequently, hearts were perfused for 50 min under conditions of left ventricular loading at a temperature of approximately 37.0 °C. Left ventricular loading was established in the *ex-situ* heart perfusion system by cannulation of the left atrium, allowing recirculating buffer to flow from the left atrium into the left ventricle and exit via the cannulated aorta. This configuration enables a working-heart setup with physiological left-sided flow. Left arterial pressure was maintained at 11.5 mmHg and aortic pressure at 60 mmHg. Immediately following the end of the experimental protocol at the various timepoints, all hearts were flushed via the aorta for three minutes with 0.9% NaCl at approximately 4 °C, followed by separation of the left ventricle, which was snap frozen for further analysis (Fig. 1).

*Ex-situ* hemodynamics (developed pressure, maximum contraction rate, maximum relaxation rate, left ventricular work (heart rate*developed pressure), left ventricular power (heart rate*developed pressure*cardiac output), triple product (heart rate*developed pressure*maximum contraction rate) were continuously monitored with the PowerLab data acquisition system (ADInstruments, Spechbach, Germany) using a micro-tip pressure catheter (Millar, Houston, USA) inserted into the left ventricle through the mitral valve. Additionally, other *ex-situ* measurements were monitored, including coronary flow, cardiac output, oxygen consumption and oxygen efficiency; all of which were reported after normalization by heart weight. Normothermic buffers were gassed with 95% oxygen and 5% CO_2_. Oxygen saturation, as well as circulating electrolytes, were measured (Cobas b 123 POC system, Roche, Rotkreuz, Switzerland) at multiple timepoints in the recirculating perfusate circuit as well as in the coronary effluent.

### Measurements of cell death markers and circulating cytokines

The release of markers of cardiac cell death was measured in samples of recirculating perfusion buffer at 60 min of reperfusion in ER-0 and ER-22 timepoints. Myoglobin and cardiac troponin I were measured by ELISA RAT MYOGLOBIN, MYO-2 and HIGH SENSITIVITY RAT CARDIAC TROPONIN I, CTNI-2-HS (Life Diagnostics Inc., Pennsylvania, USA) following the manufacturer’s instructions. Circulating cytokines were measured in serum samples collected after functional, warm *in-situ* ischemia by Bio-Plex™ Pro Rat Cytokine, Chemokine, and Growth Factor Assays (Bio-Rad Laboratories Inc., Hercules, California, USA).

### RNA Isolation and bulk RNA sequencing

Snap-frozen left ventriclar tissue of all 102 hearts include in this study were powdered and 22–30 mg tissue was used for total RNA isolation performed with the RNeasy Plus Mini Kit (Qiagen, Hilden, Germany). The total RNA was further cleaned and DNase I-treated using an RNA Clean & Concentrate-5 kit, DNase included (Zymo Research R1014) according to the manufacturer's protocol. Thereafter, the quantity and quality of the purified total RNA was assessed using a Thermo Fisher Scientific Qubit 4.0 fluorometer with the Qubit RNA BR and HS Assay Kit (Thermo Fisher Scientific, Q10211 and Q32855) and an Advanced Analytical Fragment Analyzer System using a Fragment Analyzer RNA Kit (Agilent, DNF-471), respectively. All RNA samples used for further RNA sequencing had an RNA integrity number (RIN) of 8.1–9.6.

Sequencing libraries were made using an Illumina TruSeq Stranded mRNA Library Prep kit (Illumina, 20020595) in combination with TruSeq RNA UD Indexes (Illumina, 20022371) according to Illumina’s guidelines. Pooled cDNA libraries were sequenced paired-end using a shared Illumina NovaSeq 6000 S4 Reagent Kit (300 cycles; Illumina, 20028312) on an Illumina NovaSeq 6000 instrument. This run was performed in the NovaSeq Xp workflow using a NovaSeq XP 4-Lane Kit v1.5 (Illumina, 20043131). The quality of the sequencing run was assessed using Illumina Sequencing Analysis Viewer (Illumina version 2.4.7) and all base call files were demultiplexed and converted into FASTQ files using Illumina bcl2fastq conversion software v2.20. The quality control assessments, generation of libraries and sequencing was handled by the Next Generation Sequencing Platform, University of Bern.

### Data representation and statistical analysis

#### *In-situ* and *ex-situ* heart measurements

All values for repeated measurements are reported as mean ± SD unless stated otherwise. Single measurements were compared between groups by the nonparametric Kruskal-Wallis test. If results were statistically significant (*p* < 0.05), pairwise comparisons were performed using the Dunn’s multiple comparison test. Repeated measurements of cardiac recovery were analyzed by regression analysis. P-values of cardiac recovery measurements were adjusted for multiple comparisons (modified, sequential, rejected Bonferroni procedure) [31] and considered significant if adjusted *p* < 0.05. Statistical analysis was performed with GraphPad Prism version 10 (GraphPad Software, Inc, La Jolla, USA).

#### Bulk RNA sequencing

The Bioconductor package DESeq v1.42.0 [32] was used to test for differential gene expression between groups. Gene set enrichment analysis (GSEA) [33] was run in ClusterProfiler v4.10.0 [34] using genesets from KEGG [35] (Kyoto Encyclopedia of Genes and Genomes) and MSigDb [36] (The Molecular Signature Database). An interaction term was introduced in the design formula to additionally test for differences based on sex between time-point comparisons. Principal Component Analysis (PCA) was performed on variance-stabilized transformed (VST) count data using the PCAtools R package. Variables (genes) with the lowest 10% variance were removed prior to analysis to focus on the most informative features. For the first 10 principal components, gene loadings were extracted to identify genes that contributed most strongly to sample variation. The top 30 genes with the highest absolute loading values were selected and heatmaps were generated using z-score normalized expression values. Gene set enrichment analysis (GSEA) was conducted on the ranked gene list (ordered by loading values) for each principal component using the clusterProfiler package. Enrichment was tested against KEGG pathways and MSigDB Hallmark gene sets. Statistical significance was determined using Benjamini-Hochberg adjusted p-values with a cutoff of 0.05. Weighted Gene Co-expression Network Analysis (WGCNA) was performed to identify modules of co-expressed genes and their relationships to experimental conditions using the WGCNA R package (v1.72-5). Gene expression data were obtained from DESeq2 variance-stabilized transformation (VST) of count data. For each analysis, the top 10,000 most variable genes were selected. Scale-free topology analysis was performed to determine the optimal soft-threshold power for network construction. The power achieving a scale-free topology model fit (signed R²) of ≥ 0.9 was selected. Co-expression networks were constructed using the blockwiseModules function with signed network type (default parameters). Modules were defined as clusters of highly interconnected genes with similar expression patterns across samples with a minimum module size of 20 genes. Module eigengenes (MEs) were calculated as the first principal component of each module’s expression profile. Module membership (MM) was calculated as the correlation between each gene and its module eigengene. Module–trait relationships were assessed by correlating module eigengenes with experimental variables (e.g. measurements of cardiac recovery at 60 min of reperfusion) using Pearson correlations.

Statistical significance was assessed using a Student’s t-test. For each module, two key measures were calculated: Gene Significance (GS), representing the correlation between gene expression of genes within module and traits (e.g. measurements of cardiac recovery at 60 min of reperfusion), and Module Membership (MM), representing the correlation between gene expression of genes within module and module eigengene. Hub genes were identified as the genes with the highest connectivity within each module using the chooseTopHubInEachModule function. Module gene expression patterns were visualized through mean Z-score plots across experimental conditions and comprehensive heatmaps. Functional enrichment analysis was performed for each module using multiple databases. KEGG pathway enrichment was performed using enrichKEGG and Hallmark gene sets from MSigDB were analyzed using the enricher function. All enrichment analyses used a p-value cutoff of 0.05 with Benjamini-Hochberg correction for multiple testing. All analyses were conducted in R v4.5.

#### Genome-scale metabolic pathway analysis

We performed transcriptomics-driven metabolic pathway analysis as described previously [37]. In brief, we translated the statistically significant differentially expressed genes (FDR ≤ 0.05) to enzymatic reaction rate changes according to gene–protein–reaction (GPR) rules. As a database for pathways, reactions and GPR rules, we used the Ret1 (v.1.3.0) metabolic models, genome-scale metabolic reconstruction [38]. Metabolic pathways were then scored and ranked according to the number of perturbed reactions that they encompass. We also computed p-values for each pathway to evaluate their statistical significance. We used the hypergeometric test, which is based on the hypergeometric distribution [37]. The computed p-values were subjected to a FDR correction, using the Benjamini–Hochberg procedure.

## Results

### Base characteristics

A total of 102 rats were included in this study. Detailed base characteristics are described in the Table 1 and supplementary Table S1.

**Table 1:** Base characteristics and *in-situ* hemodynamic measurements represented as median and interquartile range measured at BL; OVX, ovariectomized; HW, heart weight; BW, body weight; statistical analysis was performed by Kruskal-Wallis test followed by the Dunn`s multiple comparison test; n= 31-36 per sex

Characteristic	Females	Males	OVX
n	31	35	36
Body weight [g]	230 [210-250]^p<0.0001 vs. M and OVX^	390 [364-420]^p<0.0001 vs.OVX^	290 [271-298]
Heart weight [g]	1.1 [1.0-1.1]^p<.0001 vs. M; 0.0083 vs.OVX^	1.6 [1.4-1.9]^p<0.0001 vs. OVX^	1.2 [1.1-1.3]
HW/BW Ratio [g/kg]	4.6 [4.2-5.1]	4.3 [3.8-4.5]	4.3 [3.7-4.7]

Characteristic	Females	Males	OVX
n	25	28	31
Systolic arterial pressure [mmHg]	78.4 [74.7-80.9]^p<0.0001 vs. M and OVX^	91.9 [87.1-98.0]	78.1 [74.6-84.6]
Heart rate [bpm]	218.7 [201.2-234.7]	227.9 [213.1-245.6]	216.9 [194.8-231.2]
Pulse pressure [mmHg]	29.8 [23.5-39.6]	28.8 [15.2-36.5]	29,9 [13.1-26.7]

### Females demonstrate greater resilience to DCD-induced IRI than males

**Fig. 2:**
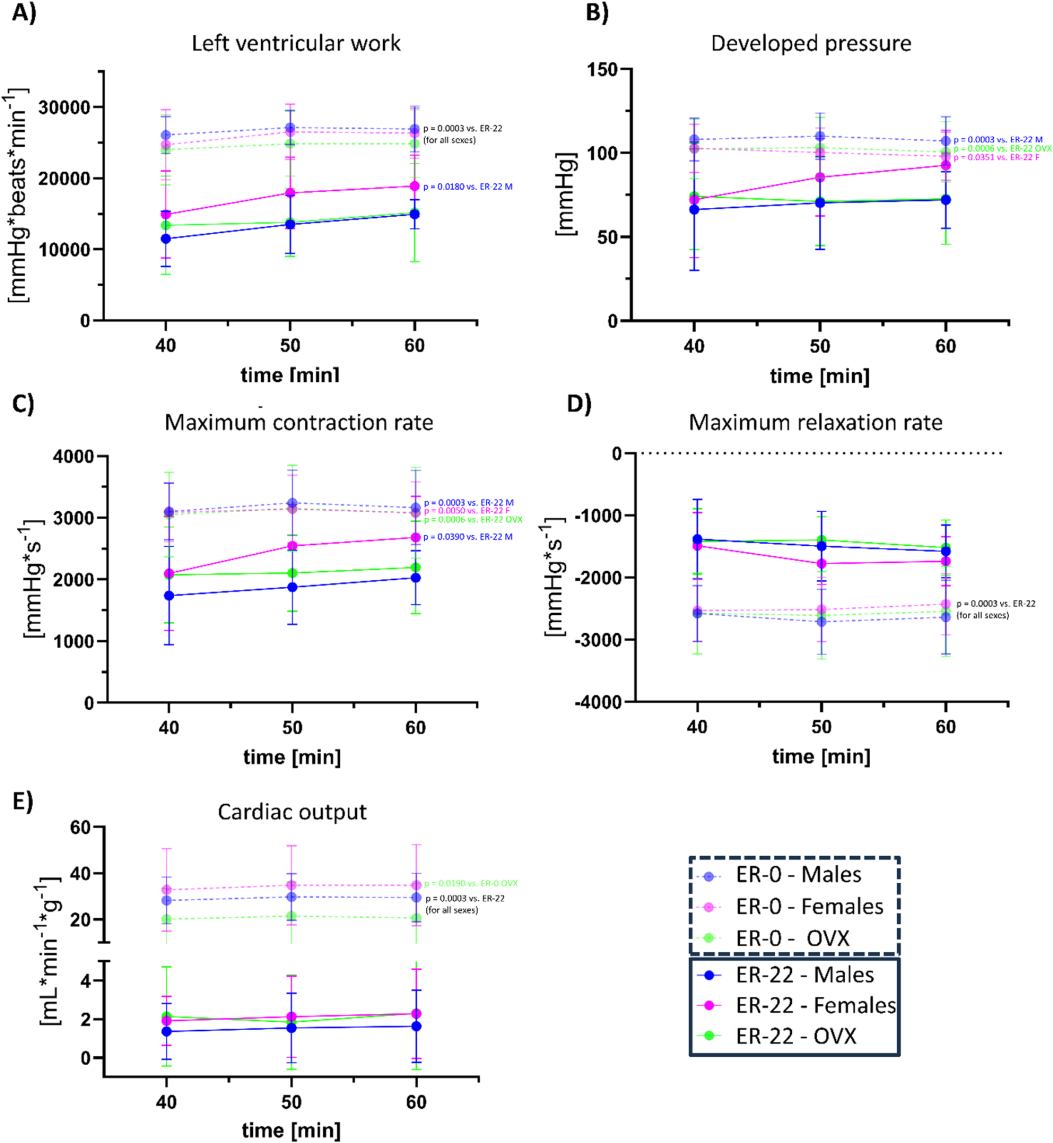
Left ventricular cardiac recovery: Cardiac recovery measurements during loaded perfusion. **A**) Left ventricular work (developed pressure*heart rate), **B**) Developed pressure, **C**) Maximum contraction rate, **D**) Maximum relaxation rate, **E**) Cardiac output normalized to heart weight; ER-0, end reperfusion 0 timepoint; ER-22, end reperfusion 22 timepoint; OVX, ovariectomized; statistical significance was evaluated by linear regression analysis and adjusted for multiple comparisons using the modified, sequential, rejective Bonferroni procedure; values are presented as means ± SD; *n* = 6–8 per group

Cardiac recovery under conditions of left ventricular loading is presented in Fig. 2. Left ventricular recovery was significantly impaired in all sexes after 22 min of functional, warm *in-situ* ischemia. In the absence of ischemia, no significant sex-related differences were detected, except for lower coronary flow in OVX animals compared with females and males (Supplementary Figure S1C).

Tolerance to DCD conditions, including IRI was significantly greater in female compared to male hearts in terms of left ventricular recovery as measured by left ventricular work (heart rate*developed pressure) and maximum contraction rate. Recovery of OVX following 22 min of ischemia and reperfusion was similar to males (Fig. 2A-E) (Supplementary Figure S1A-D).

Interestingly, markers for cardiac cell injury, myoglobin and cardiac troponin I, were not different between the sexes. However, when levels between ER-0 and ER-22 were compared, only females showed no increase in myoglobin, whereas the levels increased for both males and OVX after exposure to functional, warm *in-situ* ischemia. In addition, cardiac troponin I only increased in OVX hearts after exposure to functional, warm *in-situ* ischemia and reperfusion (Supplementary Figure S1E-F).

### Differential gene expression reveals upregulation of key pathways primarily in response to CSS and reperfusion

**Fig. 3:**
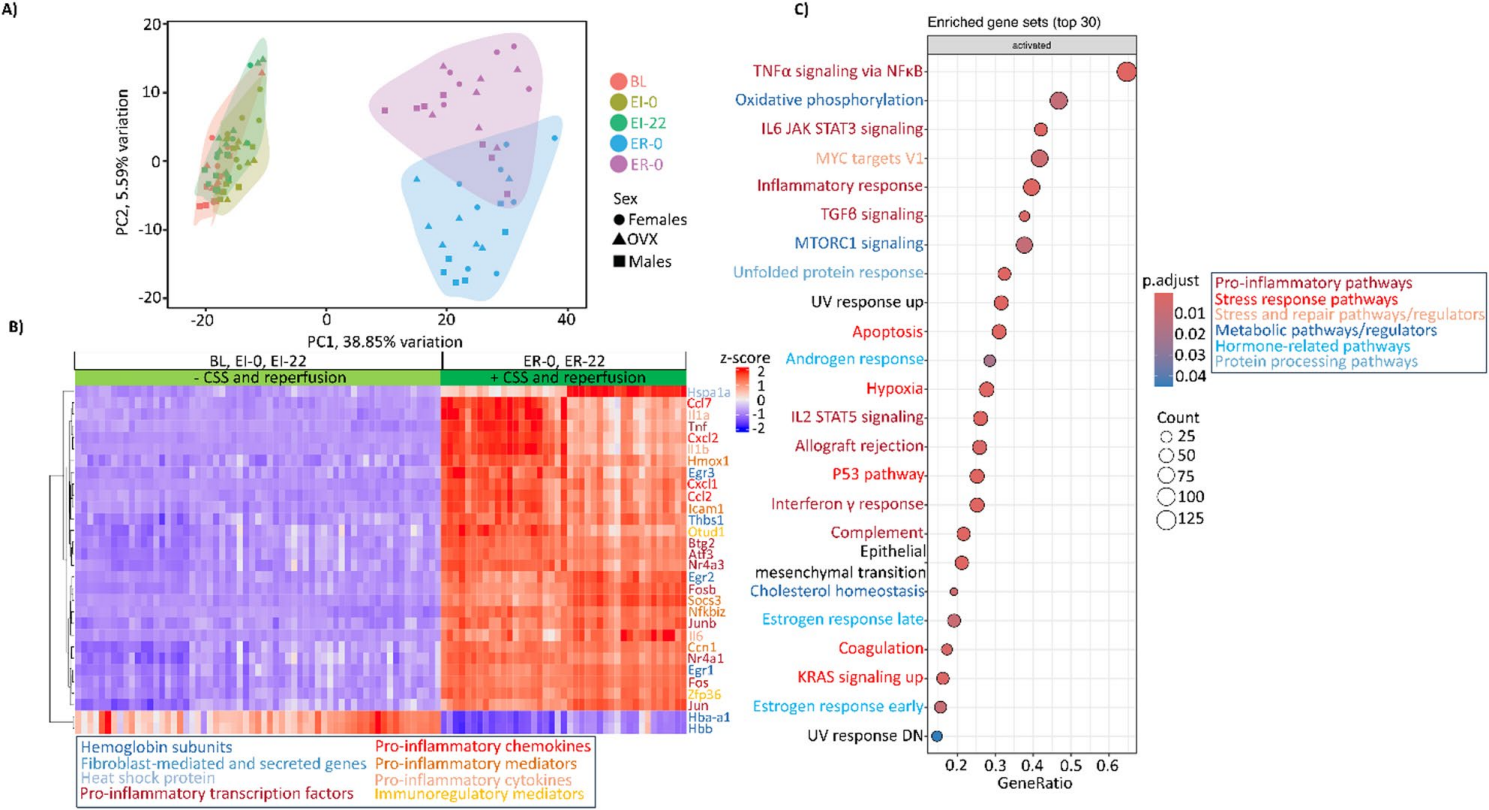
Bulk RNA Sequencing data overview: **A**) Principal component analysis including all treatment groups; **B**) Heat map representing the top 30 genes with the highest absolute loading values of PC1; **C**) GSEA (MSigDB) of genes ranked according to their loadings in the PC1. Genes and pathways are colour coded according to cellular function; BL, baseline (control timepoint); EI-0, end ischemia 0 timepoint; EI-22, end ischemia 22 timepoint; ER-0, end reperfusion 0; ER-22, end reperfusion 22 timepoint; CSS, cold static storage; GSEA, gene set enrichment analysis; MSigDB, The Molecular Signature Database; *n* = 19–23 per timepoint

To provide an overview of gene expression differences, all treatment groups were compared. This was explored by first defining the variables that most strongly contribute to the separation of groups using a principal component analysis. The identified principal component (PC1) explains nearly 40% of the variance in group separation (Fig. 3A), with segregation of hearts with reperfusion (ER timepoints) from those without reperfusion (BL and EI timepoints). Pro-inflammatory genes such as Tnf, Ccl7, Cxcl1 and 2, as well as genes that have been reported to be upregulated during cardiac mechanical stress such as Egr1 and 2 [39], were found to be strongly upregulated in reperfused hearts. Notably, Hba-a1 as well as Hbb, both genes coding for haemoglobin subunits, were found to be downregulated in reperfused hearts (Fig. 3B). Gene set enrichment analysis (GSEA) identified that genes belonging to PC1 were mainly upregulated after reperfusion and were members of pathways connected to inflammatory responses and metabolic regulation, such as TNFα signalling via NFκB and cholesterol homeostasis (Fig. 3C).

Analysis of PC2 (Supplementary Figure S2) revealed genes that contribute to the segregation of reperfusion groups with and without functional warm *in-situ* ischemia.

### Sex differences in cardiac gene expression depend on both ischemic duration and reperfusion

**Fig. 4:**
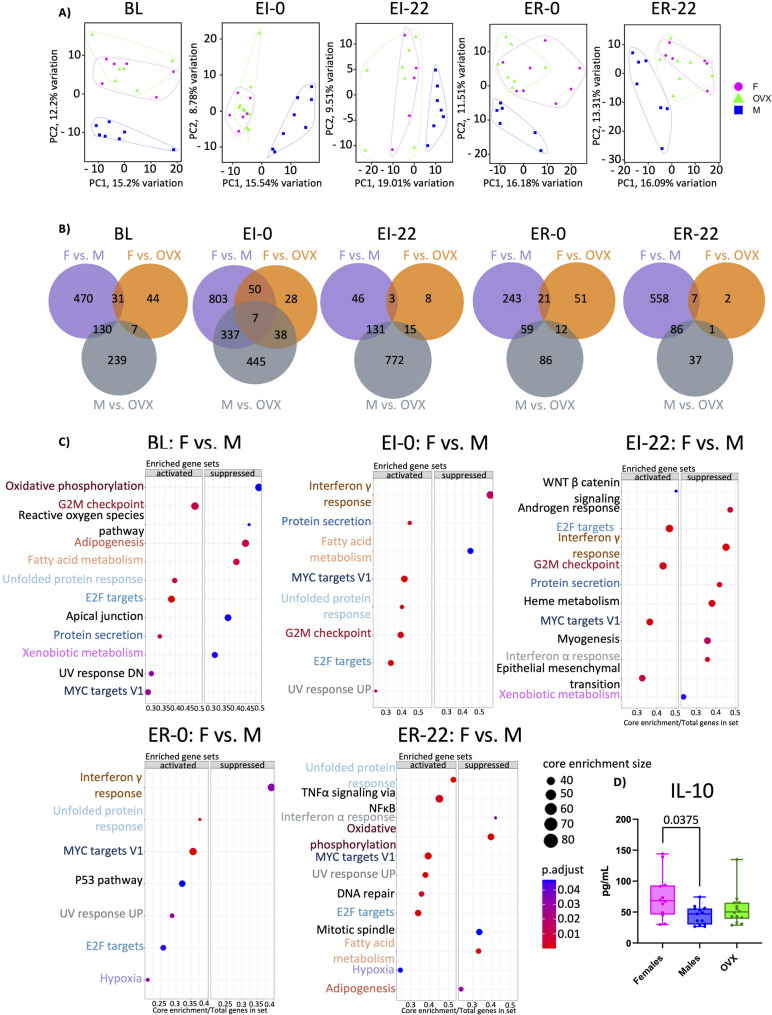
Sex differences in differential gene expression at individual timepoints: **A**) PCA for each timepoint generated by regularized log transformation showing differences in separation of sexes. **B**) Venn diagrams representing pairwise comparisons of significantly differentially expressed genes between sexes (FDR < 0.05; log2-fold change <-0.2 or > 0.2). **C**) GSEA (MSigDB) between females and males at each time point. Pathways detected multiple times are marked in the same colour, pathways depicted in black are present in only one comparison. **D**) Measurements of IL-10 at EI-22; BL, baseline (control timepoint); EI-0, end ischemia 0 timepoint; EI-22, end ischemia 22 timepoint; ER-0, end reperfusion 0; ER-22, end reperfusion 22 timepoint; Statistical significance was assessed using Student’s t-test and adjusted for multiple testing by Benjamini-Hochberg correction; GSEA, gene set enrichment analysis; MSigDB, The Molecular Signature Database; F, females; M, males; OVX, ovariectomized; FDR, false discovery rate *n* = 5–9 per group (panels **A**-**C**); *n* = 12–15 per group (panel **D**)

To reveal sex-related gene expression differences, we first analyzed each timepoint individually. Principal component analysis (PCA) at individual experimental timepoints revealed that males segregate from females and OVX rats, independent of ischemia and reperfusion, while females and OVX tend to cluster together (Fig. 4A). Overall, sex differences in gene expression persisted across DCD conditions, including IRI, with the highest number of DEGs in female–male comparisons (Fig. 4B). At each analyzed timepoint, distinct sets of genes were significantly differentially expressed between females and both males and OVX animals. To further explore which families of genes were involved, GSEA was used. This revealed that gene sets connected to E2F targets, comprising genes involved in cell-cycle regulation, as well as genes connected to MYC targets V1, which includes genes with a variety of functions, including cellular growth, repair and stress response, were activated in females versus males at all time points. Notably, genes connected to unfolded protein response were always activated in females versus males with the exception of EI-22. In general, genes connected to stress response/quality control pathways as well as regulatory aspects showed higher enrichment in females compared to males, independent of experimental timepoint. This pattern was particularly evident after 22 min of ischemia and reperfusion, where females showed enrichment not only of cell stress response/quality control pathways, but also of pathways related to DNA repair, compared with males. Gene sets related to energy metabolism, such as oxidative phosphorylation and fatty acid metabolism, showed lower enrichment in females compared with males at several timepoints (Fig. 4C). GSEA across several timepoints indicated reduced enrichment of interferon-γ pathways in females compared with males. To assess whether these transcriptomic differences were reflected at the cytokine level, a multiplex cytokine assay was performed. With the cytokine panel, we measured several cytokines at the end of functional, warm *in-situ* ischemia (IL-1α, IL-1β, IL-2, IL-4, IL-5, IL-6, IL-7, IL-10, IL-12, IL-13, IL-17, IL-18, MCP-1, G-CSF, GM-CSF, M-CSF). Among these circulating cytokines, sex-specific differences were generally not observed. However, IL-10 (Fig. 4D) and Ccl20 (data not shown) were both present at significantly higher concentrations in females compared to males after functional, warm *in-situ* ischemia.

### Pro-inflammatory signalling as well as protective pathways shape sex differences from BL to ER-22 timepoints

DEGs were compared over time, focusing on the BL to ER-22 interval for all sex groups, revealing a total of 2455 different genes. Significant differences in gene expression at ER-22 versus BL were examined within each sex to explore mechanisms of sex differences in cardiac recovery. Interestingly the majority of genes were significantly upregulated at ER-22 compared to BL in all sexes. (Supplementary Figure S3A and B).

Next, gene-expression and pathway differences between ER-22 and BL profiles were assessed and compared among the sex groups. A broad set of common pathways was detected across females, males, and OVX, including pro-inflammatory and injury-response programs such as the unfolded protein response, complement/coagulation cascades, and TGF–β–associated signalling. In contrast, transcripts linked to reactive oxygen species production were increased in only OVX at ER-22 relative to BL, whereas pathways previously associated with protective responses, most prominently DNA-repair–related genes, were downregulated exclusively in males (Supplementary Figure S3C).

Additionally, to further distinguish baseline sex differences from DCD-induced ischemic and reperfusion sex differences, an interaction term was included in the comparison of ER-22 with BL groups. Fifteen genes were identified whose expression was differentially affected by the DCD protocol in females and males, suggesting sex-specific transcriptional responses to DCD-induced functional, warm *in*-*situ* ischemia followed by reperfusion.

Notably, four of these genes already demonstrated sex-specific differences in expression at baseline (BL) that changed after reperfusion (ER-22). Ptar1, Med14, and Zkscan8 were significantly more highly expressed in females than in males at baseline. However, following the DCD protocol and subsequent reperfusion, expression levels in females tended to be lower than those observed in males. In contrast, the uncharacterized gene ENSRNOG00000065363 displayed the opposite pattern: its expression was lower in females compared with males at baseline but showed a tendency toward higher expression in females after DCD and reperfusion.

### Sex and sex hormones modulate cardiac gene expression after DCD-induced ischemia

**Fig. 5:**
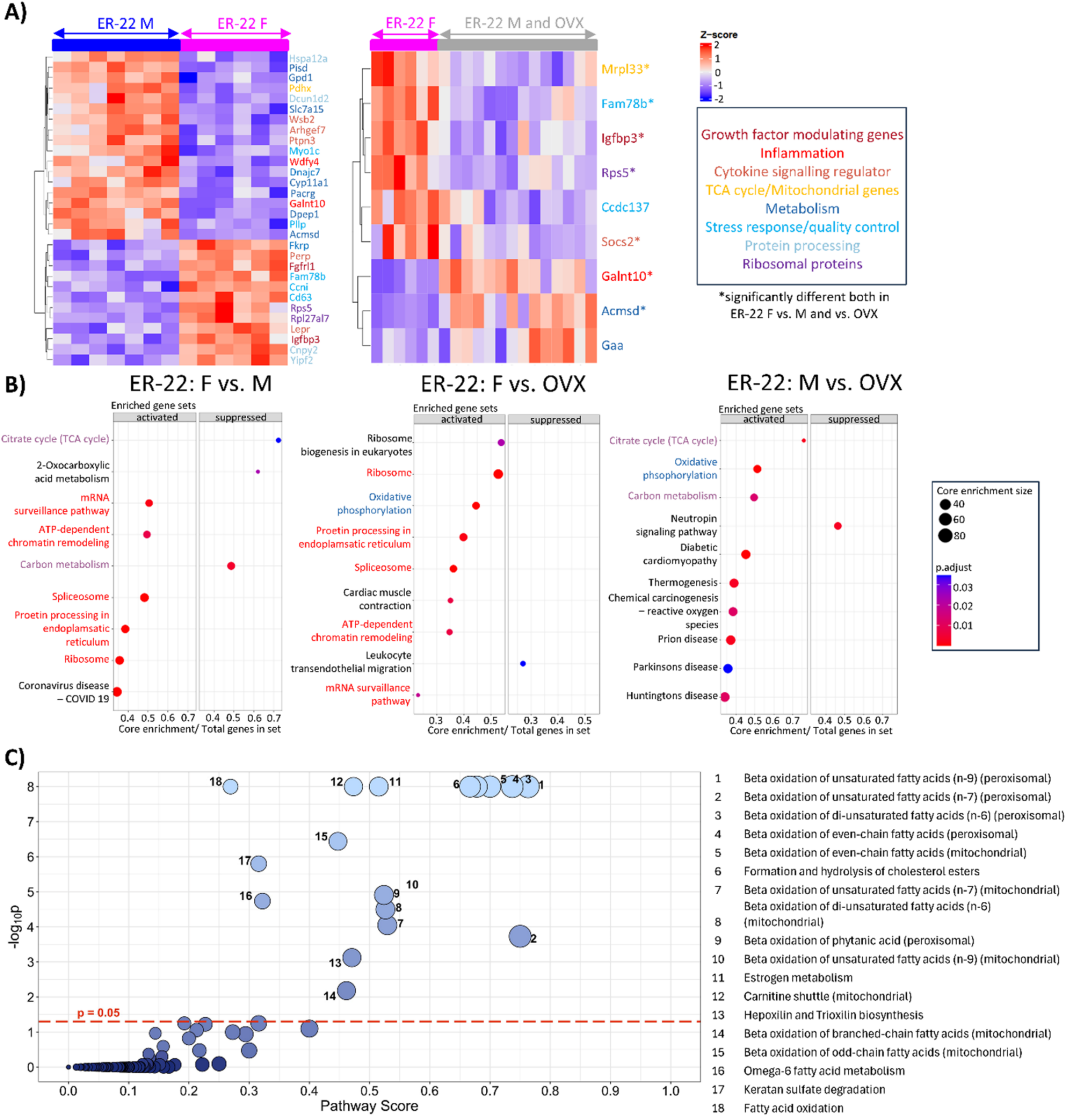
Sex differences after simulated DCD, CSS and reperfusion (ER-22): **A**) Heatmap showing top 30 differentially expressed genes, excluding X and Y chromosomal genes (FDR < 0.05; log2-fold change <-0.2 or > 0.2 ) for F versus M and for F versus M and OVX at the ER-22 timepoint, Genes are colour coded according to cellular process. **B**) GSEA (KEGG) of ER-22 F vs. ER-22 M, ER-22 F vs. ER-22 OVX and ER-22 M vs. ER-22 OVX, Pathways detected in multiple comparisons are marked in the same colour. **C**) Bubble Plot of genome scale metabolic pathway analysis of top differentially expressed pathways between females compared to males at ER-22; ER-22, end reperfusion 22 timepoint; Statistical significance was assessed using Student’s t-test and adjusted for multiple testing by Benjamini-Hochberg correction; F, females; M, males; OVX, ovariectomized; GSEA, gene set enrichment analysis; KEGG, Kyoto Encyclopaedia of Genes and Genomes; FDR, false discovery rate; Values are depicted as individual values with mean indicated; *n* = 6–7 per group

In considering only the hearts subjected to 22 min of functional, warm *in-situ* ischemia and reperfusion (ER-22 timepoint), 673 genes were found to be significantly differently expressed between females and males. Interestingly, genes related to protein processing and correct protein folding such as Hspa12a, Dcun1d and Dnajc7, were among the top 50 DEGs between males and females (Supplementary Table S2). A significant upregulation of these genes, as well as those connected to pro-inflammatory pathways such as Wdfy4 and Galnt10, was observed in males compared to females. Notably, ribosomal genes such as Rps5 and Rpl27al7 were upregulated in females compared to males at ER-22. Many genes that differed between males and females also differed between females and OVX, consistent with the pattern of ventricular functional recovery. Seven genes, including Mrpl33 and Fam78b, were downregulated in both OVX and males compared with females, and a similar pattern was observed for Rps5. Beyond these shared genes, Gaa was higher and Ccdc137 was lower in OVX compared with females (Fig. 5A and Supplementary Figure S5). Additionally, several mRNA integrity-related genes not among the top 50, including Epc1, H2az2l3, and Cdk2ap2, were downregulated in males compared with females (Supplementary Figure S6). Finally, also not among the top 50 differentially expressed genes, expression of Tfrc, a gene involved in cellular iron handling, showed significantly higher expression in males compared to females at the ER-22 timepoint.

In addition to GSEA, a genome-scale metabolic pathway analysis was performed to identify metabolic pathways differing between female and male hearts at ER-22. The main metabolic differences distinguishing females from males centre predominantly on variations of β-oxidation. Peroxisomal β-oxidation was upregulated in females, whereas mitochondrial β-oxidation was upregulated in males (Fig. 5C).

### Gene networks correlate with measurements of cardiac recovery

Weighted Gene Co-expression Network Analysis (WGCNA) was performed to define genes with similar expression patterns across samples after DCD-induced functional, warm *in*-*situ* ischemia and reperfusion. In this WGCNA, networks were defined that comprised clusters of highly interconnected genes with similar expression patterns, also referred to as modules. Further, these networks were sorted according to expression differences between the sexes, defined by functional class and correlated with measurements of functional recovery at 60 min of reperfusion.

**Fig. 6:**
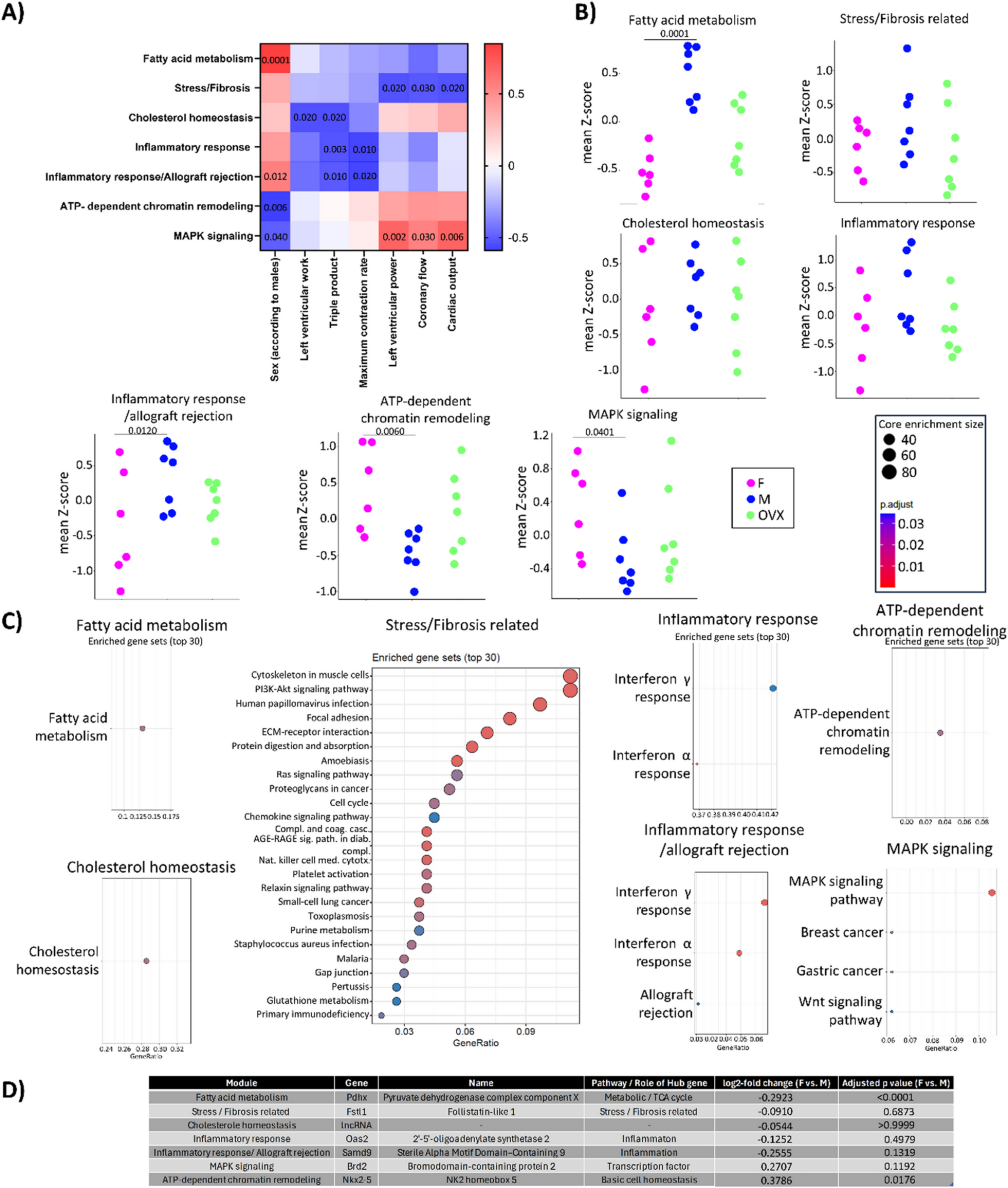
WGCNA and correlations with cardiac recovery of genes at ER-22: **A**) Correlation of networks and sex with cardiac recovery measurements (p-values depicted on corresponding correlation) **B**) Mean z-score of modules of all sexes. **C**) GSEA (KEGG) of correlated modules. **D**) Hub genes of correlated modules; Statistical significance was assessed using Student’s t-test and adjusted for multiple testing by Benjamini-Hochberg correction; Coronary flow and cardiac output measurements were normalized by heart weight; ER-22, end reperfusion 22; F, females; M, males; OVX, ovariectomized; compl., complement; coag., coagulation; casc., cascade; sig, signalling; path., pathway; diab. compl., diabetic complications; GSEA, gene set enrichment analysis; KEGG, Kyoto Encyclopaedia of Genes and Genomes; WGCNA, weighted gene co-expression network analysis; *n* = 20 (panels **A**, **C**, and **D**); *n* = 6–7 per group (panel **B**)

Genes in the MAPK (mitogen-activated protein kinase) signalling network were positively correlated with left ventricular power (heart rate*developed pressure*cardiac output), coronary flow, and cardiac output, and showed lower expression in males compared to females. Networks enriched for inflammatory responses and allograft rejection showed higher expression in males compared with females and were negatively correlated with triple product and maximum contraction rate (Fig. 6A, B and C).

For each gene network, a hub gene was defined according to the highest connectivity and interaction within the network. Interestingly, the hub genes Nkx2-5, in the ATP dependent chromatin remodeling network, and Pdhx, in the fatty acid metabolism network, were differentially expressed between females and males at ER 22, with higher Nkx2-5 expression in females and higher Pdhx expression in males (Fig. 6D).

### Genes correlating with increased cardiac recovery are upregulated in females compared to males and OVX after DCD-induced IRI

**Fig. 7:**
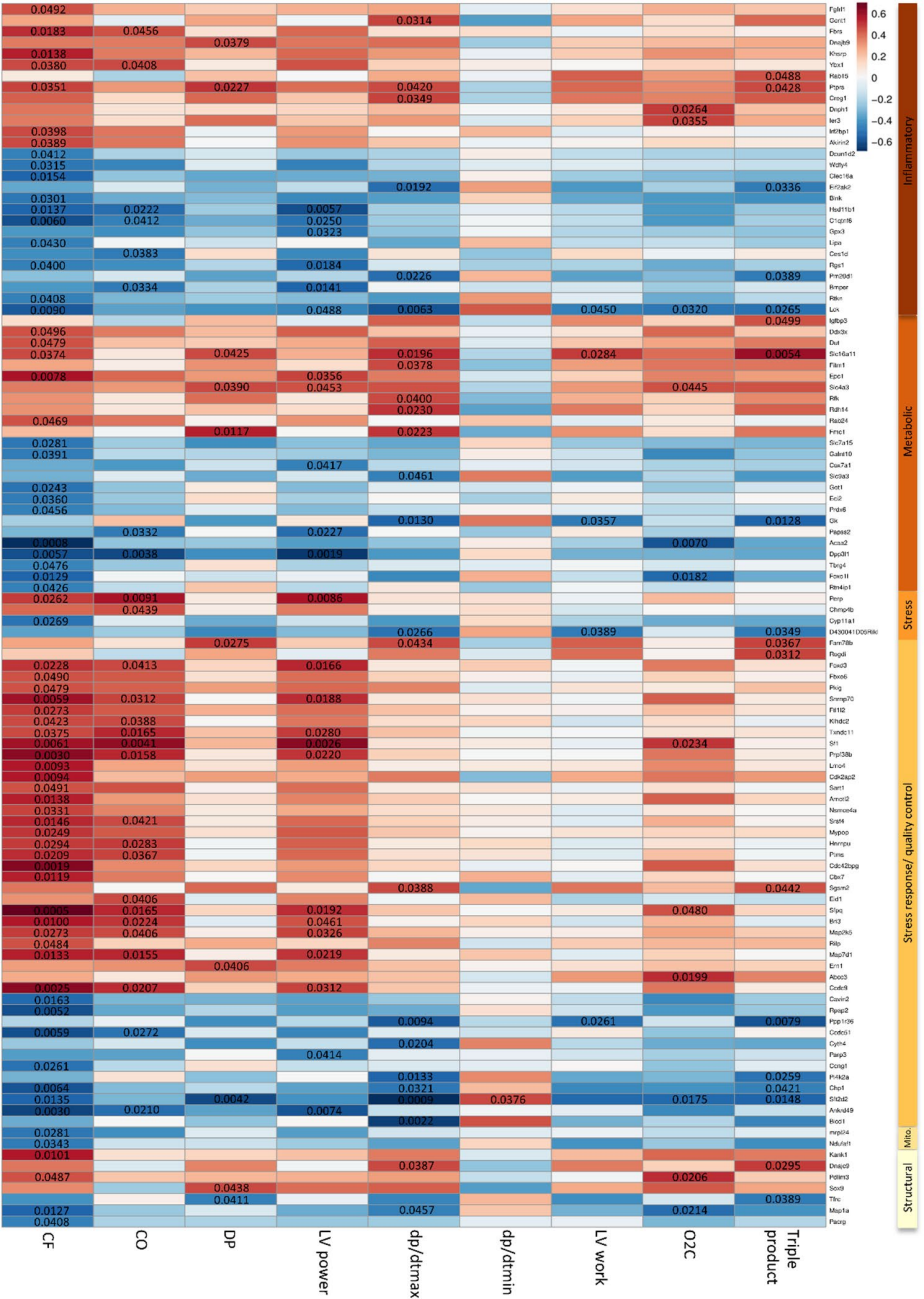
Heat map of significantly differentially expressed genes between females versus males and/or OVX correlating with cardiac recovery measurements in ER-22 hearts: R^2^ depicted from − 0.6 to 0.6; p-value depicted on corresponding correlation; statistical significance was assessed using Student’s t-test and adjusted for multiple testing by Benjamini-Hochberg correction; ER-22, end reperfusion 22; CF, Coronary flow normalized by heart weight: CO, cardiac output normalized by heart weight; DP, developed pressure; LV power, left ventricular power (heart rate*developed pressure*cardiac output); dp/dtmax, maximum contraction rate; dp/dtmin, maximum relaxation rate; LV work, left ventricular work (heart rate*developed pressure); Mito, mitochondrial; O2C, oxygen consumption normalized by heart weight; OVX, ovariectomized; *n* = 20

Expression of all genes that were significantly different for females vs. males and females vs. OVX at ER-22, were tested for correlation with measurements of cardiac recovery at 60 min of reperfusion. All genes with significantly positive correlations with cardiac recovery measurements, except for maximum relaxation rate (dp/dtmax), demonstrated a significantly higher expression in females compared to males. All genes that correlated negatively with cardiac recovery measurements, except for maximum relaxation rate (dp/dtmin), were more highly expressed in males compared to females (Fig. 7 and Supplementary Table S3).

At ER-22, seven genes, Mrpl33, Fam78b, Igfbp3, Rps5, Socs2, Galnt10 and Acmds demonstrated significantly different expression not only in females compared to males, but also in females compared to OVX. Of these seven genes, Fam78b, Igfpb3 and Galnt10 correlated significantly with at least one measurement of cardiac recovery at 60 min of reperfusion. Expression of Fam78b showed a significant positive correlation with cardiac developed pressure, maximum contraction rate and triple product at 60 min of reperfusion. A similar pattern was detected for the metabolic-related gene Igfbp3, for which expression was also significantly higher in females compared to males after DCD-induced IRI and correlated positively with the cardiac triple product at 60 min of reperfusion. Expression of Galnt10, a gene connected to metabolism and inflammatory responses, was significantly reduced after DCD-induced IRI in females compared to males. Interestingly, a high expression of Galnt10 was significantly negatively correlated with measurements of coronary flow at 60 min of reperfusion (Fig. 7).

Among the genes that were differentially expressed exclusively between females and males following DCD-induced IRI, many were associated with pathways involved in stress response/quality control. Notably, the majority of these genes demonstrated a significant positive correlation with key measurements of cardiac recovery, including coronary flow, cardiac output, and LV power at 60 min of reperfusion. In contrast, genes linked to inflammatory pathways predominantly exhibited negative correlations with coronary flow and left ventricular power (heart rate*developed pressure*cardiac output) (Fig. 7).

## Discussion

**Fig. 8:**
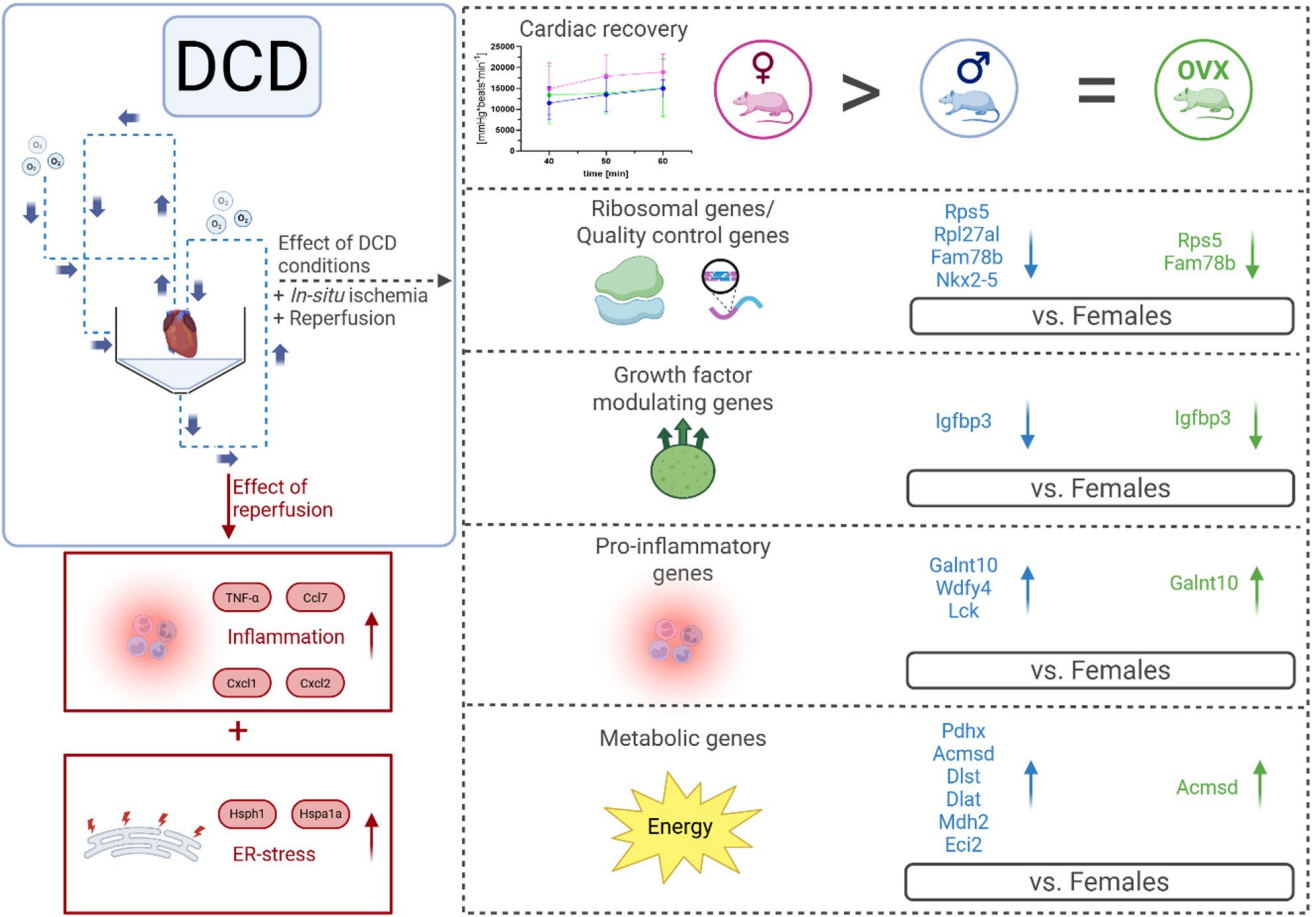
DCD conditions induce sex-specific changes in cardiac gene expression: Reperfusion induces sex-independent upregulation of stress- and inflammation- related genes. Female hearts demonstrate superior functional recovery compared with males, accompanied by increased expression of specific genes connected to quality control mechanisms and insulin-like growth factor modulation. In contrast, male and ovariectomized (OVX) hearts exhibit increased expression of specific inflammatory and metabolic genes associated with impaired cardiac recovery

In this exploratory study, we investigate left ventricular gene expression during simulation of DCD in a rat model and *ex*-*situ* cardiac reperfusion to identify transcriptomic differences that may be associated with cardiac recovery in a sex-specific manner and reveal potential new precision therapy targets. We report that ventricular function was similar in male and female hearts without functional, warm *in-situ* ischemia. Including 22 min of functional, warm *in-situ* ischemia significantly reduced recovery for all groups after 60 min of reperfusion. Notably, post-ischemic left ventricular work (heart rate*developed pressure), maximum contraction rate and triple product, measured as an additional indicator for left ventricular recovery (heart rate*developed pressure*maximum contraction rate*), were significantly higher in females compared to males, while OVX hearts closely matched those of males. This pattern of functional recovery indicates a protective role for female sex hormones. Transcriptomics analysis of all hearts demonstrated strong upregulation of inflammatory, stress response and metabolism-related pathways after reperfusion. After DCD-induced functional, warm *in-situ* ischemia and reperfusion, genes that were significantly differentially enriched in females compared with males and OVX correlated positively with cardiac recovery, whereas genes enriched in males and OVX animals versus females correlated negatively with cardiac recovery. The identification of cardiac genes and pathways that are altered in a sex-specific manner following exposure to DCD conditions provides insight into potential mechanisms of improved tolerance of female hearts to DCD conditions and may reveal novel targets for the optimization of graft quality. (Fig. 8).

Reperfusion was associated with upregulation of genes linked to endoplasmic reticulum stress and pro-inflammatory pathways, including those triggered by proteotoxic stress and misfolded protein accumulation, such as Zfand2a, Hsph1, and Hspa1a [40, 41], all of which were elevated across sex groups after 22 min of functional, warm *in-situ* ischemia and reperfusion compared to baseline. These findings align with the concept that reducing inflammatory signalling during reperfusion, for instance through steroid treatment, which is recognized to limit inflammation in DCD settings [42], could contribute to further improvement in cardiac recovery. This may be of particular benefit for male hearts, in which we demonstrate increased expression of inflammatory and allograft-rejection–related genes after IRI and reperfusion, and is consistent with reports of maladaptive remodelling and heightened inflammatory activation after left-ventricular pressure overload in males [43]. In line with these findings, GSEA showed reduced enrichment of interferon-γ–related signalling pathways in females compared with males after functional, warm, DCD-induced *in-situ* ischemia. Consistent with this pattern, independent circulating cytokine analysis revealed higher circulating IL-10 levels in females following DCD-induced ischemia. IL-10 is a well-described anti-inflammatory cytokine that has been associated with suppression of pro-inflammatory responses, including interferon-γ signalling [44–46]. Together, these observations may reflect differences in inflammatory regulation between sexes following functional, warm, DCD-induced *in-situ* ischemia.

PCA of the transcriptomics data showed a clear separation of females and males at the ER 22 timepoint and OVX hearts aligned mostly with intact female hearts, consistent with the anticipated transcriptome-wide overlap. Genes differentially expressed between males and females, include higher expression of stress-induced heat shock proteins such as Hspa12 and ubiquitin-like proteins such as Dnajc7 in males, which have been reported to be upregulated under stress-induced conditions [47, 48]. Also, pro-inflammatory genes such as Wdfy4 were more highly expressed in males compared with females only, consistent with reports in other cardiovascular diseases, including atherosclerosis [49]. Together with additional inflammatory genes such as Lck that were higher in males, these differences may be related to sex specific differences in recovery. In contrast, ribosomal genes such as Rps5 and Rpl27a were elevated in females, and higher Rps5 expression has been linked to reduced maladaptive fibrosis after simulated myocardial infarction in mice [50]. Additionally, genes involved in endoplasmic reticulum protein processing and ribosomal protein assembly were more highly expressed in females compared with males and OVX. Estrogen has been reported to suppresses maladaptive endoplasmic reticulum stress while enhancing ribosomal gene expression, thereby increasing translational capacity while preserving protein quality control [51, 52]. Additionally, GSEA revealed a post-DCD-induced IRI activation of genes related to splicosome pathways. Interestingly, the same pattern was also detected in proteomics analysis reported at the same time point after DCD-induced IRI, providing further validation of transcriptomic findings [53]. The higher enrichment of spliceosome-related genes observed in females may indicate increased RNA processing activity during cardiac stress. Alternative splicing may be dynamically regulated during myocardial injury and thereby contribute to adaptive transcriptional responses in cardiomyocytes [54]. 

Similar to our previous study, we observed sex differences with significantly higher left ventricular work (heart rate*developed pressure) and maximum contraction rate after 22 min of functional, warm *in-situ* ischemia in females versus males [15]. Findings of higher recovery of left ventricular function in females are in line with previous observations by Chen et al. [14] using a rat model of DCD, in which hearts were subjected to a comparable duration of functional, warm *in-situ* ischemia followed by *ex-situ* perfusion. Furthermore, OVX hearts showed a similar recovery to males, indicating a potential protective role of sex hormones, in line with our previous study [15].

Possible mechanisms for the increased resilience of female hearts to DCD conditions include better preserved mitochondrial integrity. We observed significantly higher expression of Fmc1 in female versus male hearts following functional, warm *in-situ* ischemia and that Fmc1 expression positively correlates with developed pressure and maximal contraction rate. As an assembly factor of mitochondrial ATP synthase (complex V), Fmc1 contributes to the maintenance of cristae architecture and oxidative phosphorylation capacity. Its increased expression in females may therefore promote improved mitochondrial integrity and bioenergetic preservation, potentially contributing to sex-specific benefits in post-ischemic functional recovery. These findings align with those of Chen et al. [14], who report reduced mitochondrial damage in female compared to male hearts in a similar model of DCD. Increased ferroptosis has also been associated with both impaired mitochondrial integrity, as ferroptotic cells display characteristic mitochondrial damage, including shrinkage, increased membrane density, and loss of cristae, and impaired recovery during machine perfusion [55, 56]. In our study, expression of Tfrc, a gene involved in cellular iron handling, was significantly higher in males compared to females only after exposure to 22 min of functional, warm *in-situ* ischemia, which may indicate a more ferroptosis-permissive milieu in males. However, Tfrc upregulation alone does not provide transcriptional evidence of ferroptosis activation and warrants further investigation of this cell death pathway. Overall, our findings indicate that improved mitochondrial integrity and function may be associated with the greater resilience of female hearts to DCD-induced warm *in-situ* ischemia.

Post-ischemic sex differences in fatty acid oxidation and metabolic flexibility may contribute to the lower cardiac recovery in males vs. females. It has been shown that upon reperfusion, high rates of fatty acid oxidation can inhibit glucose metabolism, promoting uncoupling between glycolysis and glucose oxidation and reducing contractile recovery after IRI [57, 58]. The downregulation of mitochondrial β-oxidation, together with a modest upregulation of peroxisomal β-oxidation, indicated by genome-scale metabolic pathway analysis, is consistent with a metabolic shift away from mitochondrial fatty acid oxidation in females compared to males. Such a shift may be advantageous because of a potential reduction in ROS production after IRI while maintaining partial lipid clearance via peroxisomes [59]. In contrast, higher expression of mitochondrial metabolic pathway genes in males may reflect a compensatory response to mitochondrial damage and a less efficient metabolic adaptation. These findings are in line with previous studies that showed increased resilience of mitochondrial efficiency in females compared to males, consistent with a favorable metabolic adaptation post cardiac IRI, including better coupling of glycolysis to oxidation and less acidosis, which may be related to increased cardiac recovery in females [60, 61]. 

Of particular interest after DCD-induced functional, warm *in-situ* ischemia and reperfusion, seven genes were identified as being significantly differentially expressed between both female and male hearts as well as between female and OVX hearts, a pattern that reflects post-ischemic ventricular recovery. Among these genes, Igfbp3 and Fam78b correlated positively with recovery of ventricular function, whereas Galnt10 correlated negatively with coronary flow at 60 min of reperfusion. To our knowledge, Galnt10 and Fam78b have not previously been directly linked to cardiac IRI. The shared gene expression and correlation patterns, suggest potential roles in the higher resistance of female hearts to DCD conditions, including IRI. This is consistent with a previous study by Chen et al., who demonstrated that increased Igfbp3 expression limits DNA damage and preserves cellular structure, suggesting a potential role as a cardioprotective factor in settings of cardiac injury [62]. This interpretation is further supported by our analysis of myoglobin, a marker for myocardial damage that is one of the first biomarkers to rise above normal levels after cardiac injury [63]. It was increased after ischemia in males and OVX, but not in females, indicating reduced myocardial injury in females. However, interpretation based solely on RNA sequencing data and cardiac cell damage markers may not fully explain the observed differences in recovery, and additional analyses are necessary to complement these findings. Nevertheless, the observed gene expression patterns may provide clues for potential therapeutic strategies during *ex-situ* heart perfusion. In particular, stimulation of insulin-like growth factor (IGF)-related signalling pathways associated with Igfbp3 may represent an approach to enhance cardiomyocyte survival during reperfusion, for example through supplementation with IGF-1 and activation of downstream PI3K–Akt signaling, or augmenting IGFBP3 availability in the perfusate [64]. Conversely, the negative association of Galnt10 with coronary flow suggests that targeting mucin-type O-glycosylation pathways represents another strategy to influence myocardial stress responses during organ preservation, for instance by pharmacological inhibition of polypeptide N-acetylgalactosaminyltransferase activity during *ex-situ* heart perfusion [65, 66]. Although the function of Fam78b remains largely unexplored, its consistent association with improved recovery in our dataset indicates that further investigation of the corresponding protein may help identify additional targets to enhance myocardial resilience to DCD-induced IRI.

Some significantly differentially expressed genes showed only small log2-fold changes, despite stable expression across treatment groups and sexes. This may partly reflect the short time frame of our protocol, which from baseline to end reperfusion lasted at most approximately 2 h. By selecting a relatively low log2- fold-change cutoff of 0.2, instead of the more commonly used threshold of 0.5, we aimed to avoid excluding biologically meaningful, but subtle, transcriptional changes that are common in RNA-seq datasets, particularly for regulatory or pathway effects [33]. Importantly, it has been reported that even small log2-fold changes can represent biologically meaningful differences in gene expression when supported by strong statistical significance, emphasizing that strict fold-change cutoffs may overlook relevant transcriptional changes [67, 68]. We note that statistical power in RNA-seq experiments is constrained by sample size and biological variability, which limits the reliable detection of very small expression differences. Although using a relatively low log2-fold change instead of a higher threshold increases sensitivity to subtle but potentially meaningful effects, it also raises the risk of false positives when testing thousands of genes simultaneously. To mitigate this trade-off, we controlled for multiple testing using FDR correction and focused our interpretation on consistent patterns across genes and pathways rather than isolated marginal signals. In addition, we cross-validated our selection approaches of relevant gene candidates with complementary analysis, including correlations to cardiac recovery and cytokine profiles thereby supporting the relevance of our approach despite the use of a relatively low log2-fold change threshold. Consequently, our conclusions emphasize robust, reproducible trends rather than small changes in individual transcripts.

Although our protocol is clinically relevant, several limitations should be acknowledged. Rat hearts were perfused with an oxygenated modified Krebs–Henseleit buffer rather than blood, which may influence cardiac recovery. Furthermore, only a single duration of functional, warm *in-situ* ischemia of 22 min was investigated, additional ischemic time points would allow a more precise assessment of sex-specific thresholds in ischemic tolerance. Furthermore, one cardioplegic solution (St. Thomas II supplemented with GTN and EPO) was used in this study, thus conclusions regarding the potential impact of alternative cardioplegic solutions such as Del Nido or Celsior on sex differences in cardiac recovery remain limited. As hearts were perfused *ex situ*, the contribution of primary and secondary inflammatory organs to systemic immune responses could not be fully assessed. Our experiments were limited to rat hearts; therefore, studies in larger animal models such as pigs are needed to improve translational relevance. Similarly, future studies assessing sex-specific cardioprotective responses to ischemic postconditioning in the DCD setting hold great potential to positively impact clinical protocols and graft quality.

Of key importance for the interpretation of these data, no functional perturbation experiments were performed to directly test the role of the identified candidate genes and pathways. Therefore, the mechanisms proposed to explain the observed sex differences in cardiac recovery should be viewed with caution. Although direct causal links between the identified transcriptomic differences and the improved recovery observed in female vs. male hearts cannot be established, these findings provide new insights into potential biological differences between sexes and may help guide future studies aimed at clarifying the underlying mechanisms of cardiac graft tolerance to DCD.

Also of particular relevance for this study, both perfusion duration and sampling intervals were relatively short, with the maximum experimental duration of approximately 2 h. This time frame may limit our detection of differentially expressed genes and longer time points may reveal additional changes. The short time frame of our study is connected to the relatively short half-life of many mRNA transcripts, i.e. transcriptional differences observed after 60 min of reperfusion may not exclusively represent de novo transcriptional activation, but could also reflect depletion of pre-existing transcripts or differential mRNA stability, which should be considered when interpreting early transcriptomic responses. However, the short experimental time frame may provide advantages for the identification of rapid transcriptomic changes that could contribute to early graft recovery.

## Conclusions

In conclusion, we report new information about differentially expressed genes in hearts from our established model of DCD in male, female and ovariectomized rats. We provide insights into transcriptomic changes that may help to identify potential mechanisms of DCD graft injury and therapeutic targets for tailored cardiac graft therapies. The use of *ex-situ* heart perfusion in clinical DCD heart transplantation enables new therapeutic opportunities, for example with the application of postconditioning and/or cardioprotective reperfusion strategies, that hold great potential to optimize DCD cardiac graft quality and use. Such targeted interventions could enable precision therapy for both sexes while limiting off-target toxicity, ultimately contributing to the optimization of clinical DCD protocols.

## Supplementary Information

Supplementary Material 1.

## Data Availability

The RNA-sequencing dataset generated and analysed during the current study is available in GenBank at NCBI and can be accessed with GSE317340. All additional data underlying this article will be shared on reasonable request to the corresponding authors. All data collection and animal care were performed and reported in accordance with the ARRIVE guidelines.

## Abbreviations

DCD: Donation after circulatory death
BL: Baseline
CF: Coronary flow
CO: Cardiac output
CSS: Cold static storage
DEG: Differentially expressed gene(s)
DP: Developed pressure
dp/dtmax: Maximum contraction rate
dp/dtmin: Maximum relaxation rate
EI-0: End ischemia timepoint without functional, warm *in-situ* ischemia
EI-22: End ischemia timepoint with 22 min of functional, warm *in-situ* ischemia
ER-0: End reperfusion timepoint without functional, warm *in-situ* ischemia
ER-22: End reperfusion timepoint with 22 min of functional, warm *in-situ* ischemia
F: Females
FWIT: Functional warm ischemic time
GSEA: Gene set enrichment
IGF: Insulin-like growth factor
IRI: Ischemia-reperfusion injury
KEGG: Kyoto encyclopaedia of genes and genomes
LV: Left ventricular
M: Males
MSigDB: The molecular signature database
OVX: Ovariectomized
O2C: Oxygen consumption
PCA: Principal component analysis
PC: Principal component
ROS: Reactive oxygen species
WGCNA: Weighted gene co-expression network analysis
WLST: Withdrawal of life-sustaining therapies
