# Supplementary material for: Sex differences in cardiac recovery and ventricular gene expression in a rat model of donation after circulatory death
Source: Biol Sex Differ. 2026 May 23;17:136. doi: 10.1186/s13293-026-00915-8 (PMC13386792; doi:10.1186/s13293-026-00915-8)
Supplement: Supplementary file 1 — Supplementary Material 1. [file 13293_2026_915_MOESM1_ESM.docx]

**Sex differences in cardiac recovery and ventricular gene expression in a rat model of donation after circulatory death**

Anja Helmer^1.2.3^, Alexia Clavier^1.2.3^, Maria Arnold^1.2^, Adrian Segiser^1.2^, Heidi E. L. Lischer^4.5^, Selianne Graf^1.2.3^, Mojgan Masoodi^6^, Georgia Beer^1.2.3^, Rahel Ottersberg^1.2.3^, Manuel Egle^1.2.3^, Matthias Siepe^1^, Sarah Longnus^1.2^

^1^Department of Cardiac Surgery, Inselspital, Bern University Hospital, University of Bern, Bern, Switzerland

^2^Department for BioMedical Research, University of Bern, Bern, Switzerland

^3^Graduate School of Cellular and Biomedical Sciences, University of Bern, Bern, Switzerland

^4^Interfaculty Bioinformatics Unit, University of Bern, Bern, Switzerland

^5^Swiss Institute of Bioinformatics, Lausanne, Switzerland

^6^University Institute of Clinical Chemistry, Inselspital, Bern University Hospital, University of Bern, Bern, Switzerland

**Corresponding author's contact information:**

Assoc. Prof. Dr. Sarah Longnus

Email: [sarah.henninglongus@insel.ch](mailto:sarah.henninglongus@insel.ch)

|  | BL | | | EI-0 | | | EI-22 | | | ER-0 | | | ER-22 | | |
| --- | --- | --- | --- | --- | --- | --- | --- | --- | --- | --- | --- | --- | --- | --- | --- |
|  | Females | Males | OVX | Females | Males | OVX | Females | Males | OVX | Females | Males | OVX | Females | Males | OVX |
| n | 6 | 7 | 6 | 6 | 8 | 9 | 5 | 7 | 7 | 8 | 6 | 7 | 6 | 7 | 7 |
| BW | 225 [215-258]^p=0.0004 vs. M^ | 380 [370-415] | 280 [249-290] | 237 [220-251]^p=0.0026 vs. M^ | 363 [357-390] | 290 [282-315] | 235 [215-275]^p=0.0037 vs. M^ | 415 [370-420] | 285 [263-300] | 237 [215-258]^p=0.0003 vs. M^ | 420 [368-440] | 278 [275-290] | 215 [202-231]^p<0.0001 vs. M^ | 402 [375-423] | 290 [288-300] |
| HW | 0.90 [0.5-1.2]^p=0.023 vs. M^ | 1.5 [1.4-1.5]^p=0.0122^ | 0.9 [0.9-1.1] | 1.1 [1.0-1.3] | 1.4 [1.3-1.7] | 1.1 [1.1-1.2] | 1.0 [0.9-1.3]^p=0.0059 vs. M^ | 1.8 [1.5-1.8] | 1.2 [1.1-1.3] | 1.0 [1.0-1.1]^p=0.0003 vs. M^ | 1.8 [1.6-2.0] | 1.2 [1.2-1.4] | 1.2 [0.9-1.4]^p=0.0298 vs. M^ | 1.9 [1.7-1.9] | 1.3 [1.2-1.5] |
| BW/HW | 4.43 [2.4-5.4] | 3.8 [3.5-3.9] | 3.9 [3.3-4.1] | 4.9 [4.1-5.1] | 3.7 [3.6-4.4] | 3.8 [3.6-4.2] | 4.3 [4.2-4.8] | 4.3 [4.1-4.3] | 4.3 [3.9-4.9] | 4.4 [4.2-4.8] | 4.5 [4.1-4.7] | 4.3 [4.1-5.1] | 5.6 [4.2-6.2] | 4.5 [4.2-4.9] | 4.8 [4.3-5.0] |
| PSP | - | - | - | 98.3 [85.7-103.8] | 109.6 [96.6-119.3]^p=0.0262 vs. OVX^ | 88.8 [83-8-96.5] | 100.4 [88.7-103.5] | 106.7 [99.9-123.3] | 96.3 [85.9-111.6] | 90.2 [86.0-101.4] | 107.5 [96.1-113.2] | 92.5 [87.9-100.5] | 89.0 [86.7-100.3] | 103.0 [91.3-125.7] | 97.0 [88.2-108.2] |
| MAP | - | - | - | 78.3 [73.6-28.3]^p=0.0312 vs. M^ | 94.6 [87.3-99.4]^p=0.0029 vs. OVX^ | 77.2 [72.6-82.9] | 79.3 [74.5-82.5] | 89.4 [87.1-109.5] | 82.5 [74.6-89.3] | 76.4 [73.1-80.7] | 89.6 [86.3-92.1] | 78.1 [73.3-79.5] | 78.1 [61.6-85.6]^p=0.0130 vs. M^ | 95.4 [90.7-100.3] | 81.3 [76.1-92.8] |
| PP | - | - | - | 31.7 [21.5-38.1] | 23.3 [14.4-36.7] | 26.5 [12.9-36.6] | 40.2 [37.8-45.7] | 27.4 [17.4-36.2] | 33.8 [13.1-38.4] | 28.5 [24.5-42.8] | 31.2 [23.6-38.1] | 33.5 [19.4-36.8] | 23.2 [19.9-29.2] | 20.1 [10.5-46.3] | 28.6 [12.7-37.6] |
| HR | - | - | - | 222.60 [202.8-237.9] | 232.3 [207.0-249.8] | 222.1 [198.1-231.7] | 228.6 [212.2-244.7] | 213.5 [205.7-246.4] | 216.90 [192.40-270.90] | 217.8 [200.8-235.5] | 237.8 [223.8-250.2] | 209.4 [190.5-226.8] | 205.5 [187.5-232.1] | 227.1 [184.5-243.7] | 217.1 [207.3-243.4] |

**Supplementary Table S1: Base characteristics and** ***in situ* hemodynamic measurements represented as median and interquartile range measured at BL:**

statistical analysis was performed by Kruskal-Wallis test and adjusted for multiple comparisons by Dunn’s multiple comparison test; BL, baseline (control timepoint; EI-0, end ischemia 0 timepoint; EI-22, end ischemia 22 timepoint; ER-0, end reperfusion 0 timepoint; ER-22, end reperfusion 22 timepoint; values are presented as median and interquartile range; n= 5-9 per group

**Supplementary Table S2: Top 50 significantly different expressed genes between at ER-22:**

| **Gene symbol** | **log2FoldChange** | **Adjusted p value** | **Comparison** | **Pathway** |
| --- | --- | --- | --- | --- |
| Dpep1 | -0.818410844 | 1.93E-11 | F vs. M | Metabolism |
| Slc7a15 | -0.743882344 | 2.08E-10 | F vs. M | Metabolism |
| Galnt10 | -0.527902863 | 6.21E-10 | F vs. M | Inflammation / Metabolism |
| Fam78b | 0.559684143 | 1.26E-09 | F vs. M | Housekeeping gene |
| Bex3 | 0.424004278 | 4.30E-09 | F vs. M | Inflammation |
| Igfbp3 | 0.606963039 | 2.59E-08 | F vs. M | Growth factor modulating gene |
| Fkrp | 0.257687959 | 1.27E-06 | F vs. M | Protein processing |
| Pacrg | -0.646259417 | 1.56E-06 | F vs. M | Metabolism |
| Cyp11a1 | -0.585422352 | 2.71E-06 | F vs. M | Metabolism |
| Rps5 | 0.255123605 | 2.96E-06 | F vs. M | Ribosomal |
| Pdhx | -0.292282491 | 3.24E-06 | F vs. M | TCA cycle / Mitochondrial gene |
| Fgfrl1 | 0.448567622 | 8.28E-06 | F vs. M | Growth factor modulating gene |
| Ddx3x | 0.463779767 | 8.51E-06 | F vs. M | mRNA integrity-related gene |
| Arhgef7 | -0.248916417 | 1.84E-05 | F vs. M | Cytokine signaling regulator |
| Ptpn3 | -0.365436699 | 2.01E-05 | F vs. M | Cytokine signaling regulator |
| Hspa12a | -0.358504353 | 2.36E-05 | F vs. M | Protein processing |
| Pisd | -0.252836023 | 2.36E-05 | F vs. M | Metabolism |
| Ssr4 | 0.229749477 | 2.36E-05 | F vs. M | Protein processing |
| Cnpy2 | 0.201426638 | 3.65E-05 | F vs. M | Protein processing |
| Cd63 | 0.235066575 | 3.65E-05 | F vs. M | Housekeeping gene |
| Pllp | -0.500081408 | 3.76E-05 | F vs. M | Housekeeping gene |
| Dcun1d2 | -0.260426588 | 4.68E-05 | F vs. M | Protein processing |
| Dnajc7 | -0.225925916 | 5.26E-05 | F vs. M | Protein processing |
| Gpd1 | -0.337860365 | 7.53E-05 | F vs. M | Metabolism |
| Myo1c | -0.222648747 | 8.12E-05 | F vs. M | Housekeeping gene |
| Wdfy4 | -0.555029842 | 8.12E-05 | F vs. M | Inflammation |
| Rpl27al7 | 0.367753013 | 9.74E-05 | F vs. M | Ribosomal |
| Yipf2 | 0.29539669 | 0.000100739 | F vs. M | Protein processing |
| Perp | 0.365126397 | 0.000134278 | F vs. M | Cytokine signaling regulator |
| Ccni | 0.273309178 | 0.000149837 | F vs. M | Housekeeping gene |
| Acmsd | -0.542357211 | 0.000149837 | F vs. M | Metabolism |
| Lepr | 0.511130278 | 0.000149837 | F vs. M | Growth factor modulating gene |
| Wsb2 | -0.29885992 | 0.000171405 | F vs. M | Cytokine signaling regulator |
| Nop53 | 0.270342616 | 0.000171405 | F vs. M | mRNA integrity-related gene |
| Kcnc3 | -0.48827747 | 0.000171405 | F vs. M | Ion channel/ Solute carrier-related gene |
| Acads | -0.346057912 | 0.000171581 | F vs. M | Metabolism |
| Tmem127 | -0.22429057 | 0.000171581 | F vs. M | Housekeeping gene |
| Myo16 | -0.52741481 | 0.000177522 | F vs. M | Housekeeping gene |
| Xbp1 | 0.375536863 | 0.000177853 | F vs. M | Protein processing |
| Tpcn1 | -0.247915527 | 0.000177853 | F vs. M | Ion channel/ Solute carrier-related gene |
| Ckmt2 | -0.207795285 | 0.000194713 | F vs. M | TCA cycle / Mitochondrial gene |
| Fam20b | -0.209863257 | 0.000219837 | F vs. M | Protein processing |
| Dlst | -0.212318926 | 0.000233933 | F vs. M | TCA cycle / Mitochondrial gene |
| Gria4 | 0.516293876 | 0.000258586 | F vs. M | Ion channel/ Solute carrier-related gene |
| Mecr | -0.229536012 | 0.000258586 | F vs. M | TCA cycle / Mitochondrial gene |
| Rpl7a | 0.220645867 | 0.000273548 | F vs. M | Ribosomal |

| **Gene symbol** | **log2FoldChange** | **Adjusted p value** | **Comparison** | **Pathway** |
| --- | --- | --- | --- | --- |
| Fam210b | -0.266502191 | 0.000306938 | F vs. M | TCA cycle/ Mitochondrial gene |
| Osgep | 0.335677386 | 0.000306938 | F vs. M | mRNA integrity-related gene |
| Clec16a | -0.253729373 | 0.000306938 | F vs. M | Inflammation |
| Pmm2 | -0.271898895 | 0.000313095 | F vs. M | Metabolism |
| Acmsd | -0.5867148 | 0.000603605 | F vs. OVX | Metabolism |
| Fam78b | 0.387373297 | 0.005079134 | F vs. OVX | Housekeeping gene |
| Ccdc137 | 0.374881562 | 0.01994196 | F vs. OVX | Housekeeping gene |
| Igfbp3 | 0.412580997 | 0.01994196 | F vs. OVX | Growth factor modulating gene |
| Mrpl33 | 0.238726669 | 0.034812756 | F vs. OVX | TCA cycle/ Mitochondrial gene |
| Gaa | -0.246597588 | 0.034812756 | F vs. OVX | Metabolism |
| Galnt10 | -0.30674579 | 0.047508924 | F vs. OVX | Inflammation |
| Socs2 | 0.296797544 | 0.049905161 | F vs. OVX | Cytokine signaling regulator |
| Rps5 | 0.278492646 | 0.049905161 | F vs. OVX | Ribosomal |
| FDR < 0.05; log2-fold change <-0.2 or >0.2; statistical significance was assessed using Student's t-test and adjusted for multiple testing n by Benjamini-Hochberg correction; X and Y chromosomal genes were excluded; ER-22, end reperfusion 22 timepoint; F, females; M, males; OVX, ovariectomized; n = 6-7 per group | | | | |
|  |  |  |  |  |
|  |  |  |  |  |

**Supplementary Table S3: List of potentially interesting genes based on correlation on interactome analysis:**

| **Gene symbol** | **Timepoint** | **Sex difference** | **Correlation/Interactome relationship** | **Pathway** |  |
| --- | --- | --- | --- | --- | --- |
| Srsf5 | BL | F vs. M and OVX | LV power | mRNA integrity-related gene |  |
| Nrep | BL | F vs. M and OVX | CF. HR. LV power. O2C | Housekeeping gene |  |
| Dnaja1 | BL | F vs. M and OVX | O2C | Protein processing |  |
| Dynll1 | BL | F vs. M and OVX | O2C | Housekeeping gene |  |
| Stip1 | BL | F vs. M and OVX | O2C | Protein processing |  |
| Hspa8 | BL | F vs. M and OVX | dp/dtmax. dp/dtmin. O2C. HR | Protein processing |  |
| Hsph1 | BL | F vs. M and OVX | O2C | Protein processing |  |
| Mgp | BL | F vs. M and OVX | CO. LV power | Metabolism |  |
| Pdlim3 | BL | F vs. M and OVX | CF. O2C. HR | Housekeeping gene |  |
| Rpl37a | BL | F vs. M and OVX | CO. LV power | Ribosomal |  |
| Rps29-ps15 | BL | F vs. M and OVX | CO. LV Power | Ribosomal |  |
| Cyp11a1 | EI-0 | F vs. M and OVX | CF | Metabolism |  |
| Slc7a15 | EI-0 | F vs. M and OVX | CF | Metabolism / Ion channel/ solute-carrier related gene |  |
| Slc6a8 | EI-0 | F vs. M and OVX | DP. dp/dtmax. HR | Ion channel/solute carrier- related gene |  |
| C1h9orf40 | EI-0 | F vs. M and OVX | CO | Housekeeping gene |  |
| Kctd8 | EI-0 | F vs. M and OVX | CF. O2C | Ion channel/solute carrier- related gene |  |
| Papss2 | ER-0 | F vs. M and OVX | CO. LV Power | Metabolism |  |
| Cryl1 | ER-0 | F vs. M and OVX | dp/dtmax | TCA/ mitochondrial gene |  |
| Dcn | ER-0 | F vs. M and OVX | CF. CO. LV power | Housekeeping gene |  |
| Serping1 | ER-0 | F vs. M and OVX | CF. CO. LV power | Inflammation |  |
| Ints8 | ER-0 | F vs. M and OVX | CF | mRNA integrity-related gene |  |
| **Gene symbol** | **Timepoint** | **Sex difference** | **Correlation/Interactome relationship** | **Pathway** |  |
| Gata5 | ER-0 | F vs. M and OVX | CF | Inflammation |  |
| Fam78b | ER-0 and ER-22 | F vs. M and OVX | DP. dp/dtmax. Triple product | Housekeeping gene |  |
| Igfbp3 | ER-22 | F vs. M and OVX | Triple product | Growth factor modulating gene |  |
| Galnt10 | ER-22 | F vs. M and OVX | CF | Inflammation / Metabolism |  |
| Ptar1 | BL | F vs. M | Interactome: BL vs ER-22 | Protein processing |  |
| Med14 | BL | F vs. M | Interactome: BL vs ER-22 | Housekeeping gene |  |
| Zkscan8 | BL | F vs. M | Interactome: BL vs ER-22 | Housekeeping gene |  |
| - | BL | F vs. M | Interactome: BL vs ER-22 | - |  |
| F, females; M, males; OVX, ovariectomized; BL, baseline (control timepoint); EI-0, end ischemia 0 timepoint; ER-0, end reperfusion 0 timepoint; ER-22, end reperfusion 22 timepoint; CF, coronary flow; CO, cardiac output; DP, developed pressure; LV power, left ventricular power; LV Work, left ventricular work; dp/dtmax, maximum contraction rate; dp/dtmin, minimum contraction rate; O2C, oxygen consumption; n = 6-9 per group | | | | |  |
|  |  |  |  |  |  |
|  |  |  |  |  |  |

**Supplementary Table S4: List of gene expression comparison between sexes at each timepoint with a log2-fold change <-0.5 or >0.5:**

| **Gene symbol** | **BL** | **EI-0** | **EI-22** | **ER-0** | **ER-22** |
| --- | --- | --- | --- | --- | --- |
| Dpep1 | *! | ! | *! | *! | * |
| Kdm5c | * | *! | ! | *! | *! |
| Eif2s3 |  | *! | ! | *! | *! |
| Eif2s3y | *! | *! |  | *! | * |
| Kdm5d | *! | *! |  | *! | * |
| Pbdc1 |  | *! | *! | *! | * |
| Uty | *! | *! |  | *! | * |
| Cda | *! | *! | ! |  | * |
| Kdm6a | * | * | *! | * | * |
| Klk1c3 | *! | *! | ! | * |  |
| Obp3 |  | *! | *! | * | * |
| Pacrg |  | ! | *! | * | * |
| Rgs17 | *! | *! | ! | ! |  |
| Crlf1 | * | $! | ! | ! |  |
| Ms4a4c-ps1 | *! | *! |  | * |  |
| Slc7a15 | * | * | *! |  | * |
| Stmn4 |  | *! | ! | ! | * |
| Atp4a | * |  | *! |  | * |
| Ces1dl1 |  | *! | *! |  |  |
| Ddx3x |  |  |  | *! | *! |
| Hmcn2 | * | * |  | * | * |
| LOC103694537 | *! |  |  | *! |  |
| Lepr | * |  |  | *! | * |
| Nptx1 |  | $! | *! |  |  |
| Ptprt | * | * | * | * |  |
| Slc9a3 | *! | * |  |  | * |
| Wdfy4 |  | * | *! |  | * |
| Adamts15 | ! | *! |  |  |  |
| B3gat1 | * |  |  | *! |  |
| C1qtnf6 | * | *! |  |  |  |
| Ces1e |  | *! | ! |  |  |
| Cyp11a1 |  | *$ |  |  | * |
| Ddx3y | ! | ! |  | ! |  |
| Phf24 | * | * |  |  | * |
| Unc79 | ! | * | ! |  |  |
| Adh1c |  | *! |  |  |  |
| Alas2 |  |  | *! |  |  |
| Atp6v1g2 |  |  |  | *$ |  |
| Blnk |  | *! |  |  |  |
| Chadl |  |  | ! |  | * |
| Cxcl9 |  |  |  | * | * |
| F13a1 |  | $! |  |  |  |
| Fgf9 |  |  | ! | ! |  |
| Fkbp5 |  |  | *! |  |  |
| Frmpd1 |  |  | *! |  |  |
| **Gene symbol** | **BL** | **EI-0** | **EI-22** | **ER-0** | **ER-22** |
| Gbp1 |  |  |  | *! |  |
| Hsd11b1 |  | ! |  | * |  |
| Igf1 |  | *! |  |  |  |
| Igfbp3 | * |  |  |  | * |
| Il15 |  | *! |  |  |  |
| LOC100912991 |  |  |  | *! |  |
| Lgals5 |  |  | *! |  |  |
| Lrrc17 |  |  | *! |  |  |
| Mfap2 |  | *! |  |  |  |
| Ms4a6b |  |  |  | *! |  |
| Orm1 |  |  | *! |  |  |
| Pde4b | * | * |  |  |  |
| Pdlim2 |  | *! |  |  |  |
| Pla2g2a |  | *! |  |  |  |
| Plau |  | *! |  |  |  |
| Rbm44 | * |  |  |  | * |
| Slc16a11 |  |  |  | * | * |
| Tdrd1 |  | * |  | * |  |
| Trh |  | *! |  |  |  |
| Vegfd |  | $! |  |  |  |
| Abcg1 |  |  | ! |  |  |
| Abra | * |  |  |  |  |
| Ache |  |  |  | * |  |
| Ackr1 |  | ! |  |  |  |
| Acmsd |  |  |  |  | * |
| Acta1 |  |  |  | * |  |
| Adcy1 |  |  |  |  | * |
| Adra1d | ! |  |  |  |  |
| Akap3 |  | * |  |  |  |
| Aldh1a3 |  | * |  |  |  |
| Aox3 |  | ! |  |  |  |
| Atp1a3 |  |  | ! |  |  |
| Atp8a1 | * |  |  |  |  |
| Bex3 |  |  |  |  | * |
| C1qa |  | ! |  |  |  |
| C1qb |  | ! |  |  |  |
| C1qc |  | ! |  |  |  |
| C1qtnf7 |  | ! |  |  |  |
| C7 |  | ! |  |  |  |
| Capn6 |  |  | ! |  |  |
| Ccdc80 |  | ! |  |  |  |
| Ccn1 |  |  |  | * |  |
| Cd300c2l1 |  | ! |  |  |  |
| Ces1c |  |  | ! |  |  |
| **Gene symbol** | **BL** | **EI-0** | **EI-22** | **ER-0** | **ER-22** |
| Ces1d | * |  |  |  |  |
| Cfh |  | ! |  |  |  |
| Clec4a1 |  | ! |  |  |  |
| Coch |  |  | ! |  |  |
| Col8a1 |  |  |  |  | * |
| Cox7a1 |  |  |  |  | * |
| Cpm |  |  |  |  | * |
| Cpxm2 |  |  | ! |  |  |
| Crhr2 |  | * |  |  |  |
| Crispld2 |  | ! |  |  |  |
| Cthrc1 |  |  | ! |  |  |
| Dixdc1 |  |  |  | ! |  |
| Dlg2 |  | ! |  |  |  |
| Dusp13b |  |  | ! |  |  |
| Dusp15 |  |  | ! |  |  |
| Dynll1 | * |  |  |  |  |
| Egr1 |  |  |  | * |  |
| Epn3 | * |  |  |  |  |
| Fam78b |  |  |  |  | * |
| Fcer1g |  | ! |  |  |  |
| Fcna |  | ! |  |  |  |
| Fgf2 | * |  |  |  |  |
| Fgfrl1 |  |  |  |  | * |
| Fmo3 |  | ! |  |  |  |
| Folr2 |  | ! |  |  |  |
| Fxyd2 |  | ! |  |  |  |
| Gabre |  | ! |  |  |  |
| Galnt10 |  |  |  |  | * |
| Gcnt1 |  |  |  |  | * |
| Gpr63 |  | * |  |  |  |
| Gpr68 |  |  |  | * |  |
| Gpr88 | * |  |  |  |  |
| Gria4 |  |  |  |  | * |
| Hba-a2 |  |  | * |  |  |
| Hmgn5 |  |  |  | * |  |
| Hopx |  |  |  |  | * |
| Ints6l | * |  |  |  |  |
| Kcnc3 |  |  |  |  | * |
| Kcnip3 | * |  |  |  |  |
| Kcnj14 |  | * |  |  |  |
| Kctd8 |  | * |  |  |  |
| Klk15 |  | ! |  |  |  |
| Krt2 | * |  |  |  |  |
| **Gene symbol** | **BL** | **EI-0** | **EI-22** | **ER-0** | **ER-22** |
| LOC102556096 |  | ! |  |  |  |
| LOC120103526 |  |  |  | * |  |
| Ldlr |  |  |  | * |  |
| Lrrn2 |  | * |  |  |  |
| Ltbp2 |  |  | ! |  |  |
| Map1a |  |  |  |  | * |
| Mfap3l |  |  |  |  | * |
| Mrc1 |  | ! |  |  |  |
| Ms4a4a |  | ! |  |  |  |
| Mt2A |  | ! |  |  |  |
| Mtcl1 |  |  |  |  | * |
| Mthfd2 |  | ! |  |  |  |
| Myo16 |  |  |  |  | * |
| Nat8f3 |  | ! |  |  |  |
| Nenf |  |  |  |  | * |
| Nr4a3 | * |  |  |  |  |
| Nup210l |  | * |  |  |  |
| Ogdhl |  |  | ! |  |  |
| P4ha1 | * |  |  |  |  |
| Pamr1 |  |  | ! |  |  |
| Pgf |  | * |  |  |  |
| Pgpep1l |  | * |  |  |  |
| Phyhd1 |  |  | ! |  |  |
| Pllp |  |  |  |  | * |
| Prdm1 |  |  | * |  |  |
| Ptgds |  |  | ! |  |  |
| Rbp7 |  |  | * |  |  |
| Ret | * |  |  |  |  |
| Rin1 |  |  |  |  | * |
| Rnase1 |  |  |  | * |  |
| Rsrp1 | * |  |  |  |  |
| Sbsn |  | * |  |  |  |
| Scamp5 |  | ! |  |  |  |
| Sdk1 |  | ! |  |  |  |
| Sel1l3 |  | * |  |  |  |
| Septin3 |  | $ |  |  |  |
| Sh3bp2 |  |  |  | * |  |
| Siglec1 |  | ! |  |  |  |
| Slc15a2 |  | ! |  |  |  |
| Slc43a2 |  |  |  |  | * |
| Slc43a3 |  | ! |  |  |  |
| Spint2 |  |  |  |  | * |
| Ssmem1 |  | * |  |  |  |
| **Gene symbol** | **BL** | **EI-0** | **EI-22** | **ER-0** | **ER-22** |
| St8sia2 |  |  | ! |  |  |
| St8sia5 |  | * |  |  |  |
| Tgm3 | $ |  |  |  |  |
| Tlr7 |  | ! |  |  |  |
| Tmem150c |  |  |  |  | * |
| Vipr2 |  |  |  | ! |  |
| Wfdc1 |  | * |  |  |  |
| Ypel3 |  |  |  |  | * |

F, females; M, males; OVX, ovariectomized; BL, baseline (control timepoint); EI-0, end ischemia 0 timepoint; ER-0, end reperfusion 0 timepoint; ER-22, end reperfusion 22 timepoint; *, significantly differentially expressed in females compared to males; !, significantly differentially expressed in females compared to OVX; $, significantly differentially expressed in males compared to OVX = 5-9 per group

Supplementary figures:


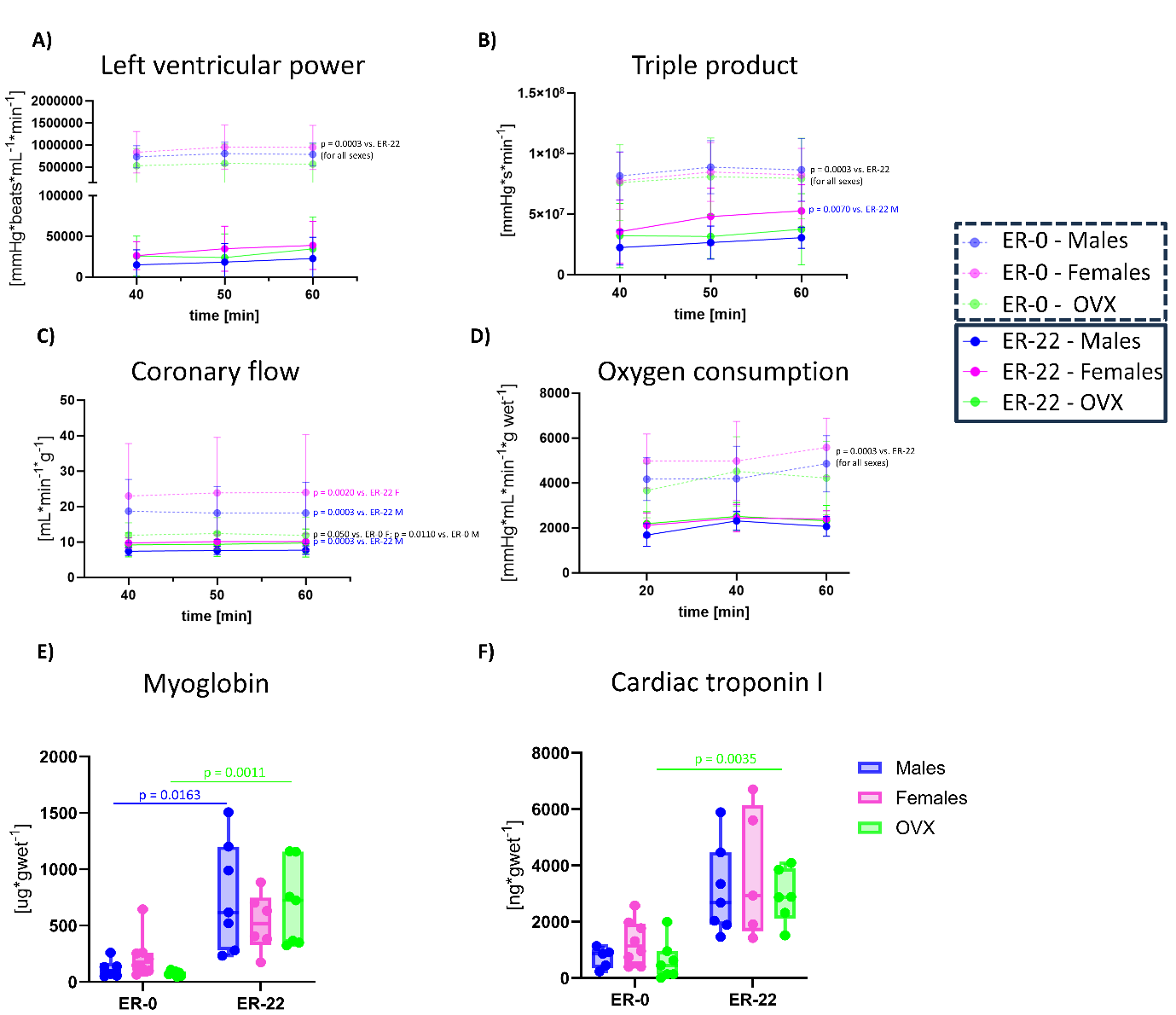


**Supplementary Figure S1: Left ventricular cardiac recovery and release of myocardial cell death markers myoglobin and cardiac troponin I during reperfusion:** Cardiac recovery measurements during loaded reperfusion. A) Left ventricular power (heart rate*developed pressure*cardiac output). B) Triple products. C) Coronary flow normalized by heart weight. D) Oxygen consumption normalized by heart weight. E) Myoglobin in recirculating perfusate after 60 minutes of reperfusion in timepoints ER-0 and ER-22 in females. males and OVX rats. F) Cardiac troponin I in recirculating perfusate after 60 minutes of reperfusion in timepoints ER-0 and ER-22 in females, males and OVX rats; ER-0, end reperfusion 0 timepoint; ER-22, end reperfusion 22 timepoint; OVX, ovariectomized; statistical analysis of cardiac recovery was performed by linear regression analysis and adjusted for multiple comparisons by the modified, sequential, rejective Bonferroni procedure; values are presented as means ±SD;  statistical analysis of cell death markers was performed by Kruskal-Wallis test followed by Dunn’s multiple comparison test; values are presented as median with interquartile range; n = 6-8 per group


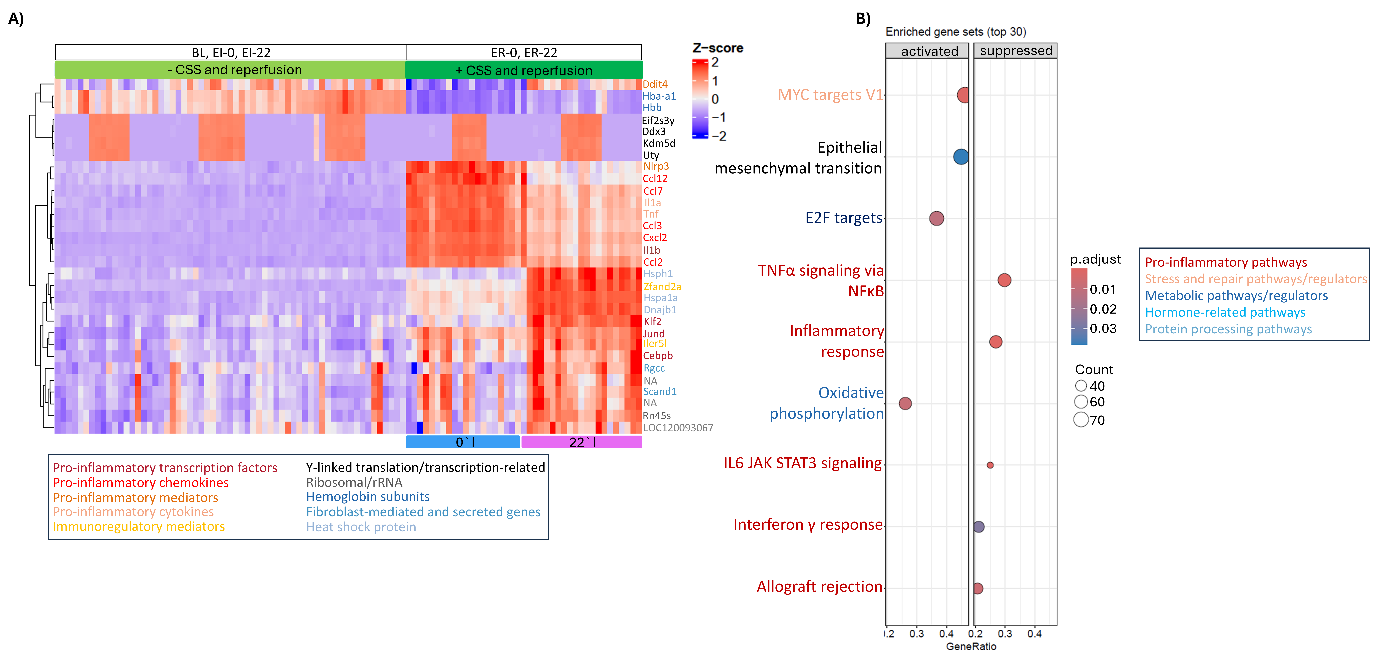


**Supplementary Figure S2: Bulk RNA Sequencing data overview.** A) Heat map representing the top 30 differentially expressed genes of PC2. B) Gene set enrichment analysis of differentially expressed genes in PC2; genes and pathways are colour coded according to cellular process; BL, baseline (control timepoint); EI-0, end ischemia 0 timepoint; EI-22, end ischemia 22 timepoint; ER-0, end reperfusion 0 timepoint; ER-22, end reperfusion 22 timepoint; CSS, cold static storage; GSEA, gene set enrichment analysis; MSigDB, The Molecular Signature Database; n= 19-23 per timepoint


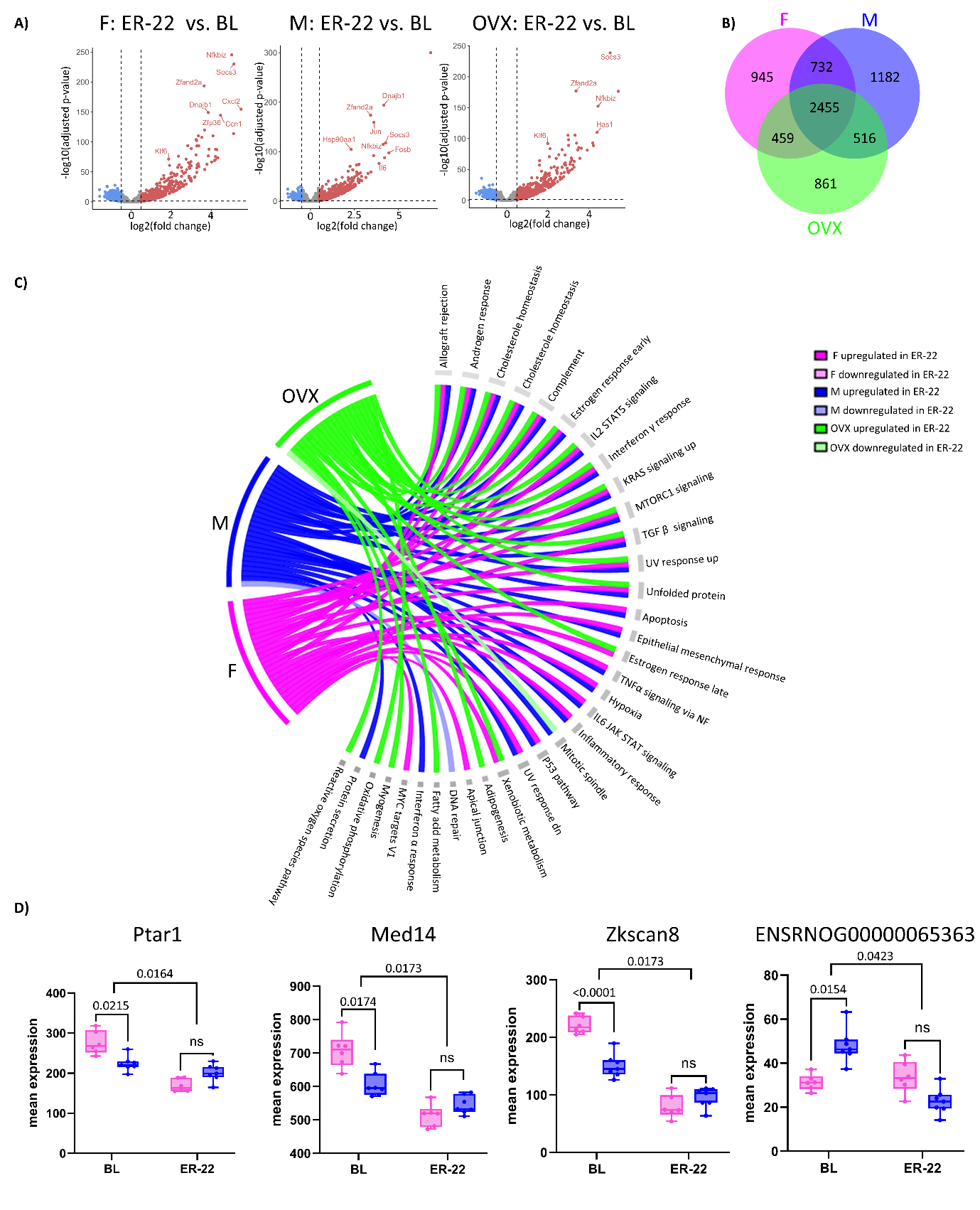


**Supplementary Figure S3: Comparison of differential gene expression between ER-22 and BL:** A) Volcano plots of differentially expressed genes between ER-22 and BL for females. males and OVX (FDR <0.05; log 2-fold change > 0.5 or < -0.5). B) Venn diagram of differentially expressed genes (all genes are represented as up-regulated) between ER-22 and BL of all three sexes. C) GSEA chord plot of all MSigDB pathways significantly differentially expressed between ER-22 and BL timepoint D) Genes differentially expressed based on interactome analysis between ER-22 and BL; Statistical significance was assessed using Student's t-test and adjusted for multiple testing by Benjamini-Hochberg correction; BL, baseline; ER-22, end reperfusion 22 timepoint; GSEA, gene set enrichment analysis; MSigDB, The Molecular Signature Database; F, females; M, males; OVX, ovariectomized; FDR, false discovery rate; n= 19-20 per timepoint (panel A); n= 6-7 per group (panels B-D)


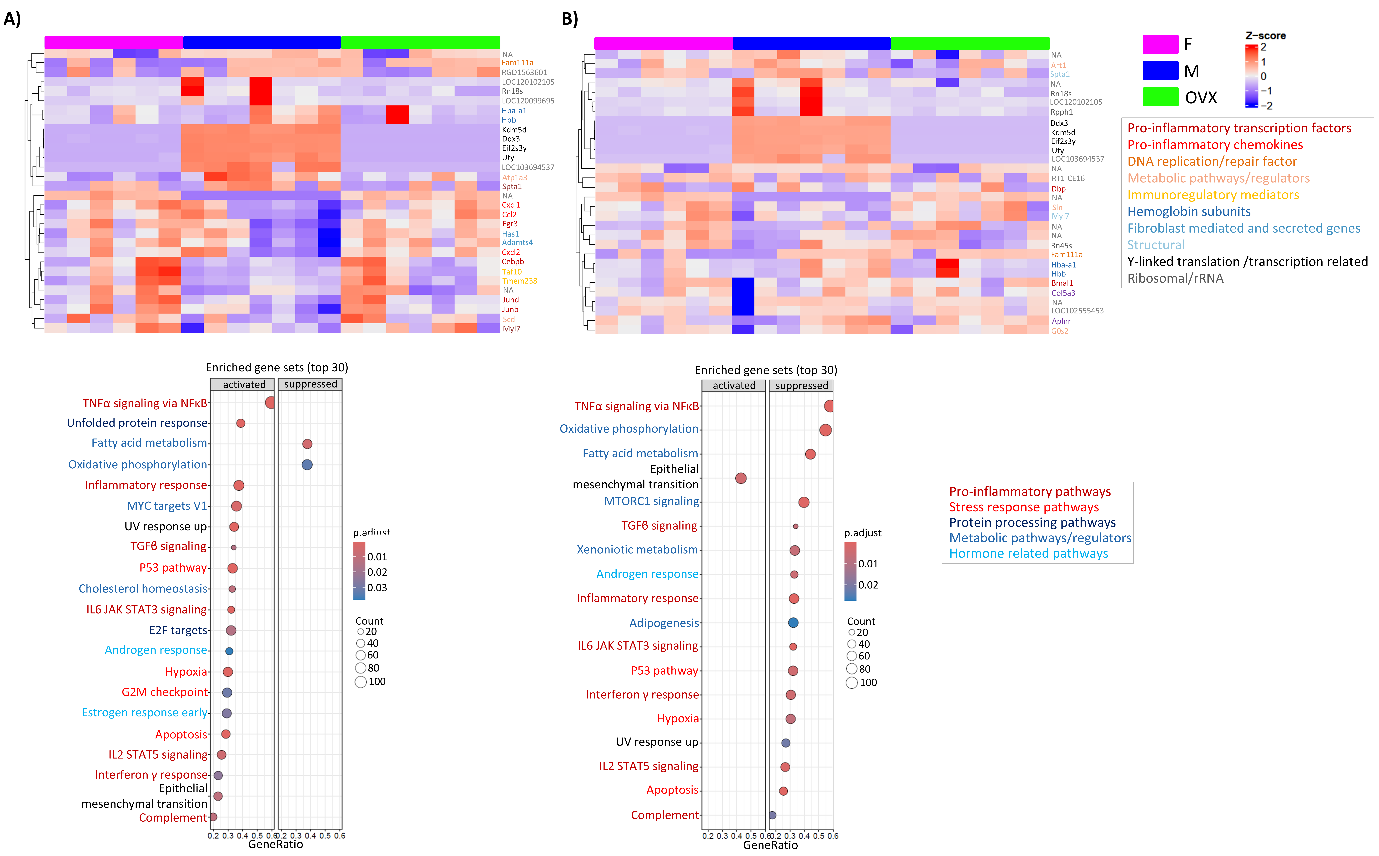


**Supplementary Figure S4: Sex differences at ER-22: Sex differences after simulated DCD. CSS and reperfusion:** A) Heat map representing the top 30 differentially expressed genes of PC1 and GSEA (MSigDB) of PC2. B) Heat map representing the top 30 differentially expressed genes of PC2 and GSEA (MSigDB) of PC2; genes and pathways are colour coded according to cellular process; ER-22, end reperfusion 22 timepoint; F, females; M, males; OVX, ovariectomized; CSS, cold static storage; DCD; donation after circulatory death; GSEA, gene set enrichment analysis; MSigDB, The Molecular Signature Database; n= 6-7 per group


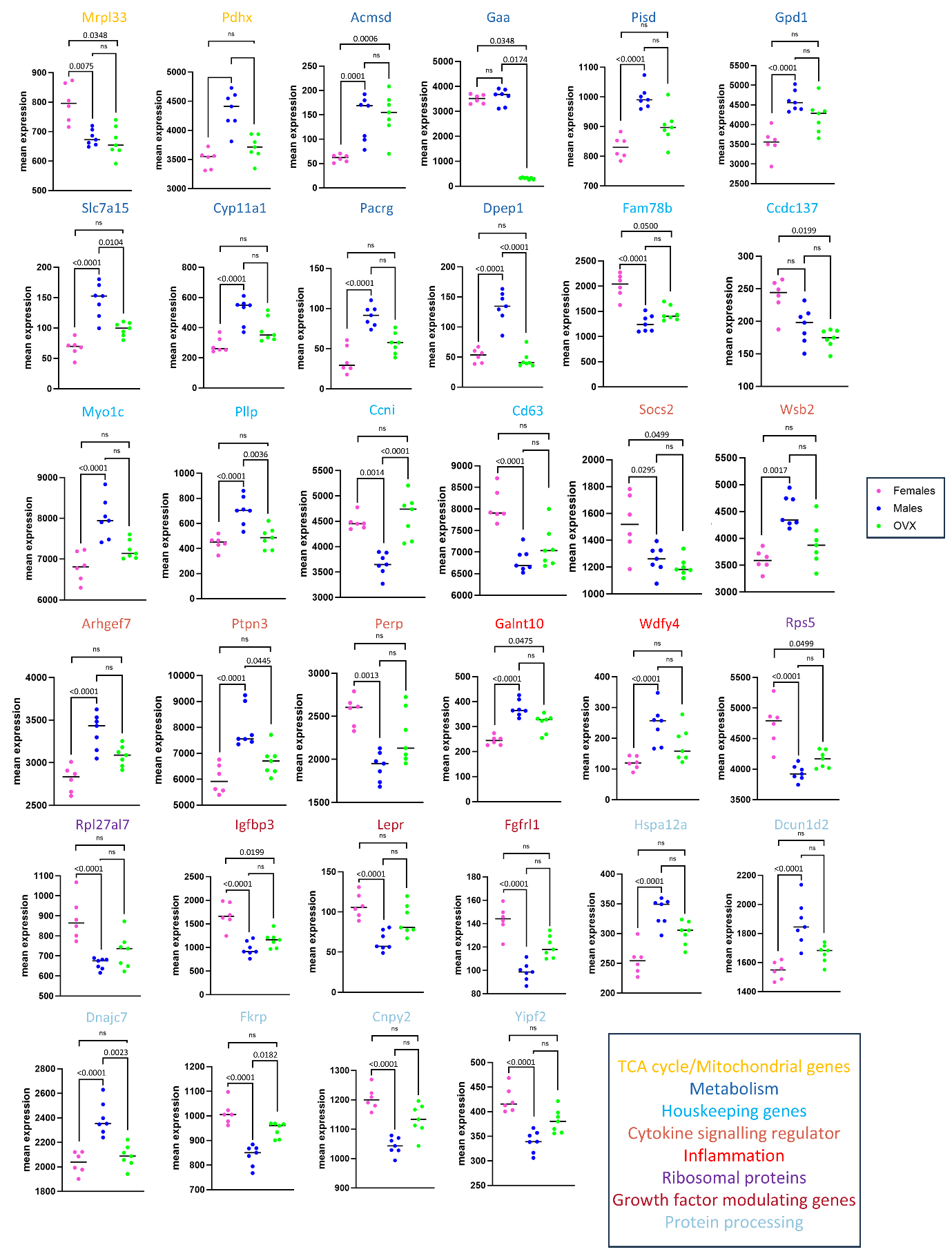


**Supplementary Figure S5: Sex differences at ER-22: Sex differences after simulated DCD. CSS and reperfusion:** Normal expression values of top 30 differentially expressed genes (FDR < 0.05; log2-fold change <-0.2 or >0.2 ); Genes and pathways are colour coded according to cellular process; Statistical significance was assessed using Student's t-test and adjusted for multiple testing by Benjamini-Hochberg correction; ER-22, end reperfusion 22 timepoint; F, females; M, males; OVX, ovariectomized; CSS, cold static storage; DCD; donation after circulatory death; Values are depicted as individual values with mean indicated; n= 6-7 per group


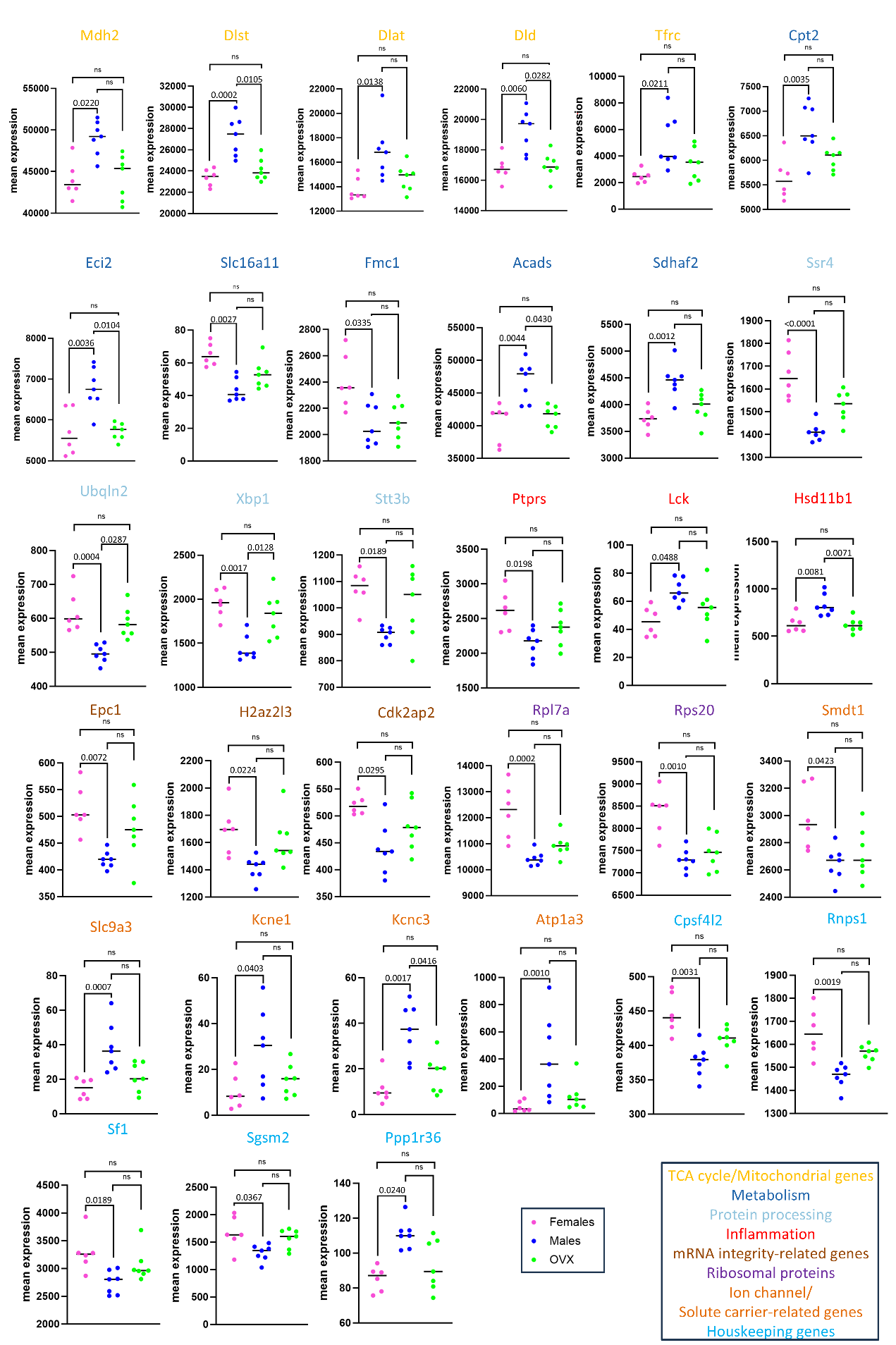


**Supplementary Figure S6: Sex differences at ER-22: Sex differences after simulated DCD. CSS and reperfusion:** Normal expression values of significantly differentially expressed genes between females and males after DCD induced IRI (FDR < 0.05; log2-fold change <-0.2 or >0.2 ); Genes and pathways are colour coded according to cellular process; Statistical significance was assessed using Student's t-test and adjusted for multiple testing by Benjamini-Hochberg correction; ER-22, end reperfusion 22 timepoint; F, females; M, males; OVX, ovariectomized; CSS, cold static storage; DCD, donation after circulatory death; Values are depicted as individual values with mean indicated; n= 6-7 per group
